# Prioritization and functional assessment of noncoding variants associated with complex diseases

**DOI:** 10.1186/s13073-018-0565-y

**Published:** 2018-07-11

**Authors:** Lin Zhou, Fangqing Zhao

**Affiliations:** 1 Computational Genomics Lab, Beijing Institutes of Life Science, Chinese Academy of Sciences, Beijing, 100101 China; 2 University of Chinese Academy of Sciences, Beijing, 100049 China; 3 Center for Excellence in Animal Evolution and Genetics, Chinese Academy of Sciences, Kunming, 650223 China

**Keywords:** Complex disease, Functional annotation, Genetic variant, Variant prioritization, Noncoding variant

## Abstract

Unraveling functional noncoding variants associated with complex diseases is still a great challenge. We present a novel algorithm, Prioritization And Functional Assessment (PAFA), that prioritizes and assesses the functionality of genetic variants by introducing population differentiation measures and recalibrating training variants. Comprehensive evaluations demonstrate that PAFA exhibits much higher sensitivity and specificity in prioritizing noncoding risk variants than existing methods. PAFA achieves improved performance in distinguishing both common and rare recurrent variants from non-recurrent variants by integrating multiple annotations and metrics. An integrated platform was developed, providing comprehensive functional annotations for noncoding variants by integrating functional genomic data, which can be accessed at http://159.226.67.237:8080/pafa.

**Electronic supplementary material:**

The online version of this article (10.1186/s13073-018-0565-y) contains supplementary material, which is available to authorized users.

## Background

Recent advances in sequencing technologies have enabled the identification of an increasingly large spectrum of variants within the human genome [1]. However, unraveling the genetic architecture of complex diseases is still a great challenge, particularly identifying functionally relevant variants in noncoding regions [2, 3]. Previous studies have interpreted coding variants based on our understanding of the genetic code and splicing [4]. Many existing computational approaches have been developed for prioritizing these variants, such as SIFT [5] and PolyPhen [6]. Noncoding variants, however, are noticeably understudied due to our poor understanding of noncoding regions in the human genome. Most recently, tremendous progress has been achieved in both large-scale functional genome projects (e.g., ENCODE [7] and FANTOM5 [8]) and human genome resequencing projects (e.g., 1000 Genomes Project [9]), which provide a rich resource of genomic annotations for analyzing and predicting the functional effects of both coding and noncoding variants.

Recently, several computational approaches, including both unsupervised and supervised algorithms, have been developed to prioritize noncoding variants by integrating various genomic features, including functional annotations and evolutionary conservation. To prioritize risk variants, unsupervised statistical methods (e.g., GenoCanyon [10] and Eigen [11]) construct discriminative models based on conditional probability distributions, which rely on strong model assumptions. Supervised methods (e.g., CADD [12], FATHMM series [13–15], DANN [16], GWAVA [17], and DIVAN [18]) do not rely on a priori assumptions; instead, they label the training data as deleterious or benign and fit a model that best separates the two sets. These integrative supervised methods generally outperform those based on any single individual feature [11, 12] and frequently provide more than one score depending on the regions considered (e.g., coding, noncoding) and the appropriate feature sets for that region. The scores, however, sometimes may lead to conflicting evaluation results for variants. Besides, some of these methods have intrinsic limitations in prioritizing specific categories of risk variants. For example, CADD constructed a model based on the training variants that have been under long-term selective pressure, which made it perform less well on certain disease-associated variants under weak evolutionary constraint, such as those influencing the risk of complex traits [11, 12]. LINSIGHT [19] was constructed based on the premise of inferring the selective pressure on noncoding sites and worked very well on identifying human noncoding variants associated with inherited diseases; however, this premise may not hold in all cases, such as those in which the variants increase the risk for post-reproductive diseases [19]. In addition, except for genomic annotations and conservation measures, all the currently available methods seldom consider population-level statistical measures (e.g., *F* statistics [20]), which may be helpful to prioritize common variants. Although supervised learning demands a representative and correctly labeled training set, a major problem for these methods is the use of mislabeled variants in the training stage, which may lead to false predictions by supervised classifiers. For example, DIVAN labeled variants from the 1000 Genomes Project as benign with few controlling or filtration steps. A considerable fraction of the variants in the 1000 Genomes is reported to be involved in various complex diseases or traits [21, 22]. CADD labeled fixed or nearly fixed derived alleles in humans as benign and simulated de novo variants as deleterious. However, such simulated de novo variants may contain a substantial proportion of benign variants, which thus may lead to false predictions.

Here, we present a novel supervised algorithm for Prioritization And Functional Assessment (PAFA) of genetic variants associated with complex diseases or traits, especially for population-specific noncoding variants. PAFA can prioritize functional variants in noncoding regions by utilizing all kinds of available annotations and metrics, including genomic annotations, evolutionary conservation metrics, and population level measures. In particular, a newly introduced feature, *F*_ST_, which is frequently used as a summary of genetic differentiation among groups [20], can significantly help PAFA prioritize population-relevant functional variants in noncoding regions over background variants. In addition, to obtain more reliable training variants, PAFA utilizes training data from various curated databases, and it employs multiple filtration strategies for variant labeling. Through comprehensive evaluations of both common and rare variants, we demonstrate that PAFA exhibits a much better performance on prioritizing both common and rare complex disease-associated variants over benign variants as well as discriminating between noncoding recurrent variants and non-recurrent variants through the incorporation of multiple features and the optimization of training datasets. Moreover, a user-friendly web server (http://159.226.67.237:8080/pafa) was constructed that not only allows users to evaluate variants by PAFA but also provides comprehensive functional annotations by integrating abundant functional genomic elements.

## Methods

### Data and annotation sources

**Fig. 1:**
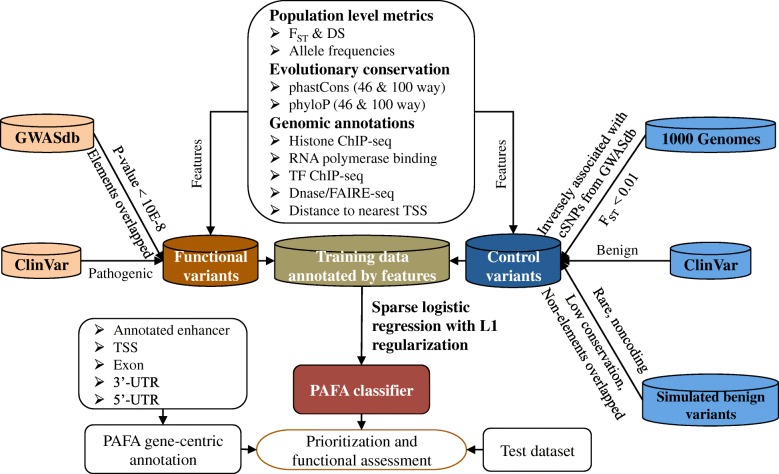
Flowchart of the PAFA approach. The flowchart contains the construction of the PAFA classifier and the gene-centric annotation. The PAFA classifier is based on sparse logistic regression with L1 regularization. We label the variants used in the training stage of PAFA as the functional and control sets. The features used in PAFA include three categories: population-level metrics, evolutionary conservation, and genomic annotations. Gene-centric annotation is based on curated genomic databases, including ENCODE and UTRdb

Genetic and genomic resources used to construct and validate the PAFA algorithm are mainly divided into three categories (Fig. 1 and Additional file 1: Figure S1). Firstly, PAFA selected variants from the 1000 Genomes Project (Phase 3) [9], ClinVar (released in 2018/3/1) [23], and GWASdb (v2) [24] as the training set of PAFA. The functional variant dataset included variants labeled “pathogenic” in ClinVar and significant SNPs associated with complex traits or diseases (cSNPs) that overlap with known genomic elements from GWASdb. Correspondingly, variants labeled “benign” in ClinVar and variants in 1000 Genomes were treated as a control dataset. The calculation of PAFA scores is based on the GRCh37/hg19 human genome assembly, as the new genome build (GRCh38) still lacks enough genomic annotations compared with GRCh37. Here, we integrated a lift-over tool [25] for users who choose GRCh38 as the reference.

Secondly, PAFA selected annotations from known databases as features to annotate training variants and to evaluate new variants. These features can be divided into three classes: evolutionary conservation metrics, genomic annotations, and population differentiation measures. For evolutionary conservation, two measures, phastCons [26] and phyloP [27], were obtained. Conservation scores based on the comparison of both 46 and 100 vertebrate genomes were used. For genomic annotations, PAFA used both genic context information, such as distance to nearest transcript start site (TSS) from GENCODE v19 annotation [28] and information from thousands of functional genomic elements across different cell types, including histone modifications, RNA polymerase binding, and transcription factor binding sites (TFBS PeakSeq). For population differentiation measures, *F*_ST_ and dispersion score (DS) were calculated based on allele frequencies and sample sizes of the five super populations. Based on the coding and noncoding annotations, we also built a gene-centric database to provide gene-level annotations for variants. To determine which variants may affect gene expression, we retrieved annotated exons and transcription start site information from GENCODE v19 [28] and 5′-UTR and 3′-UTR data from UTRdb [29]. The predicted enhancers that regulate the target genes were also obtained [30]. In addition, we recorded intron regions that are overlapped with any annotations, such as open chromatin and transcription factor binding sites (TFBS), from ENCODE [31]. With this integrated genomic annotation system, PAFA can link a variant to known genes or genomic elements.

Thirdly, we used test sets from seven public databases (the 1000 Genomes Project Phase 3, ClinVar, GWAS Catalog [32], COSMIC v79 [33], TCGA [34], GRASP v2.0 [35], and ICGC [36]) and variants from three recent studies including 916 breast cancer variants [37], 221 human blood metabolites variants [38], and 1764 macular telangiectasia type 2 variants [39], to compare PAFA with other available methods (Additional file 1: Figure S1). We used five different types of variant sets from four databases to perform a comprehensive evaluation, including benign and pathogenic coding variants of ClinVar, common variants with frequencies of at least 1% in the population studied in 1000 Genomes, complex diseases or trait-associated SNPs (cSNPs) of the GWAS Catalog, and recurrent noncoding variants of COSMIC. Then, we used Mendelian disease-associated variants of ClinVar and complex disease-associated variants from 560 breast cancer samples in a recent study and TCGA database to compare their performance on prioritizing coding risk variants. We used variants of GRASP, 221 variants associated with human blood metabolites, and 647 common variants associated with macular telangiectasia type 2 to assess PAFA’s performance in prioritizing noncoding variants associated with complex diseases or traits. Finally, we used variants from ICGC Cancer Genome Projects to assess PAFA’s ability in discriminating both common and rare recurrent from non-recurrent variants and in prioritizing noncoding rare risk variants from adjacent common variants.

For the PAFA online platform, it was integrated with several additional utilities, including 1000 GENOMES, ANNOTATION, VSEA, and SEARCH (Additional file 1: Figure S2–5). Due to the population-scale sequencing feature of variants in the 1000 Genomes Project, the 1000 GENOMES part of the online platform utilized the population differentiation index and allele frequency of a specific variant among human populations to assist the prioritization and annotation of target variants. Correspondingly, except for known variants and genomic resources mentioned above, these utilities were also integrated with other resources. First, the ANNOTATION part of online platform integrated variants from dbSNP [40], NHLBI-Exome Sequencing Project v2 (ESP) [41], YanHuang Project [42], CNV of 100 pancreatic ductal adenocarcinomas (PDACs) [43], schizophrenia somatic deletions in brain [44], and 996 ASD rare CNVs [45] to annotate input variants. With these annotated resources, we can determine whether the input variants overlap with known variants through the online platform. Simultaneously, the ANNOTATION part also integrated with a variety of curated databases containing disease-associated genes, including Online Mendelian Inheritance in Man (OMIM) [46], the Genetic Association Database (GAD) [47], and COSMIC [33], to recognize potential disease-related variants. In addition, the VSEA part incorporated canonical pathways from the Molecular Signatures Database (MSigDB v6.1) [48], to perform enrichment analysis on variants based on these included pathways.

In total, 23 curated genetic and genomic resources were integrated into the PAFA online platform (Additional file 1: Figure S6), including known variants, various annotations, disease-associated genes, and pathways. All annotations are represented in the GRCh37 assembly of the human genome.

### Construction of functional and control variant sets in PAFA

The training set of PAFA was mainly derived from curated databases, including ClinVar, GWASdb, and 1000 Genomes. Considering that these public databases may contain redundancies and erroneous or conflicted records, we employed multiple filtrations to remove low-confidence variants (Additional file 1: Figure S7). We firstly eliminated conflicted records in the training set that are labeled “benign” and “pathogenic” in ClinVar at the same time. A possible explanation is that these variants do not cause certain diseases, but they may contribute to the development of diseases in other cases [49, 50]. Therefore, they were removed from the control dataset, but they were kept in the functional dataset. In total, 30,277 “pathogenic” and 13,010 “benign” variants from ClinVar were included in PAFA. Twenty four thousand nine hundred ninety-three cSNPs from GWASdb were selected as extremely significant variants using a threshold of *p* ≤ 10E−8. After removing cSNPs that share no overlap with known genomic elements, 11,570 variants from GWASdb were used in PAFA. To select non-functional variants from 1000 Genomes, we randomly selected 100,000 variants from 1000 Genomes with a low *F*_ST_ (< 0.01) along with the filtration of redundant records in GWASdb and constructed a regression model for cSNPs in the training set on the basis of 7131 evaluation features. L1-regularized logistic regression, which is provided by LIBLINEAR [51], was used to construct the model. Using the constructed regression model, we determined the numerical measures of these 100,000 variants with little genetic differentiation. We ranked these variants according to their numerical output, as negative values mean variants inversely associated with cSNPs in the training set. According to their ranking, 28,837 inversely associated variants were selected as a control dataset by PAFA.

As described above, we introduced variants from ClinVar, GWASdb, and 1000 Genomes, including pathogenic/benign coding variants and common functional/benign variants. Ultimately, 41,847 functional and 41,847 control variant datasets were used by PAFA (Additional file 1: Figure S7).

### Selection and analysis of features in PAFA

Based on the existing classifiers, PAFA first pre-selected 7131 features that may be sensitive to noncoding variants, which can be divided into three classes, including conservation metrics, genomic annotations, and population differentiation measures. We introduced four evolutionary conservation scores, including 46 and 100 ways of phastCons and phyloP measures. For genomic annotations, we introduced eight types of feature groups from ENCODE [7], including histone modifications (ChIP-Seq), RNA contigs (Long RNA-seq), transcription factor binding sites (TFBS PeakSeq and SPP), open chromatin (DNase-Seq and FAIRE), and transcript start site (TSS).

For population differentiation measures, we introduced allele frequencies of five super populations, including African, American, East Asian, European, and South Asian. We calculated *F*_ST_ and dispersion score (DS) based on allele frequencies and sample sizes of the five super populations. For a given genomic locus, consider *i* subpopulations (where *i* = 1, …, *s*) and suppose that the observed allele frequencies are *p*1, …, *ps* and the sample sizes are *n*1, …, n*s*. Let $n={\sum}_{i=1}^s{n}_i$ and $\overline{n}={\sum}_{i=1}^s{n}_i/ s$.

Wright’s unbiased *F*_ST_ [52] is estimated as

$$ {F}_{\mathrm{ST}}=\frac{MSP- MSG}{MSP+\left({n}_c-1\right)\times MSG} $$

where MSG denotes the observed mean square errors for loci within populations

$$ MSG=\frac{1}{\sum_{i=1}^s{n}_i-1}\sum \limits_i^s{n}_i{p}_i\left(1-{p}_i\right) $$

and MSP denotes the observed mean square errors for loci between populations,

$$ MSP=\frac{1}{s-1}\sum \limits_i^s{n}_i{\left({p}_i-\overline{p}\right)}^2 $$

with $\overline{p}$as a weighted average of *p*_*i*_ across populations

$$ \overline{p}={n}_i{p}_i/{\sum}_i{n}_i $$

and *n*_*c*_ is the average sample size across samples that also incorporates and corrects for the variance in sample size over populations.

$$ {n}_c=\frac{1}{s-1}\sum \limits_{i=1}^s{n}_i-\frac{\sum_i{n}_i^2}{\sum \limits_i{n}_i} $$

The dispersion score is calculated as

$$ \mathrm{DS}=\sqrt{\frac{\sum_{i=1}^s{\left({p}_i-\overset{=}{p}\right)}^2}{n}} $$

with

$$ \overset{=}{p}=\sum \limits_{i=1}^s{p}_i/s $$

We constructed feature vectors for variants. These features had fixed unique sequence numbers. We performed tenfold cross-validation with the training set mentioned above to assess the pre-selected features in PAFA, including four conservation scores, seven types of feature groups for genomic annotations, and three population differentiation measures (Additional file 1: Figure S8). All annotation feature groups employed by PAFA have the ability to prioritize functional variants from a control set, with AUC values larger than 0.5. Thus, PAFA adopted all these features to annotate variants.

### Model training and performance comparison

With a mass of instances and features, PAFA employed LIBLINEAR [51] to construct an ensemble discrimination model against variants. LIBLINEAR is an efficient and open source library for large-scale linear classification. PAFA treated features of a variant without an overlapping relationship as missing values and took L1 regularization to construct a sparse model. PAFA adopted the logistic regression implemented in LIBLINEAR, which was used to calculate the probability PAFA scores for variants.

To evaluate the performance of PAFA in prioritizing functional variants, seven widely used classifiers were compared with PAFA, namely, CADD, FATHMM-MKL, DANN, GWAVA, DIVAN, LINSIGHT, and Eigen. CADD has updated three versions since its publication. Here, the latest version of CADD was used to generate *C* scores for variants. By using different genomic annotations, FATHMM-MKL provided two different scores, namely, a “coding score” and a “noncoding score,” which were deemed to prioritize coding and noncoding variants, respectively. We used both scores for comparison. Based on different training sets from 1000 Genomes, GWAVA provided three independent scores, Region, TSS, and Unmatched, which were all used for performance comparisons. Similar to GWAVA, DIVAN also provided Region and TSS scores. Considering that DIVAN provided disease-specific scores for SNPs associated with 45 diseases or phenotypes, PAFA was compared to DIVAN on discriminating these disease- or phenotype-related variants. Moreover, Eigen provided two scores for evaluating variants by using different algorithms. To compare with Eigen, we downloaded the Eigen scores of the testing sets from its website (http://www.columbia.edu/~ii2135/download.html) and also compared with their pre-computed Eigen and Eigen-PC scores. In addition, we also obtained testing sets from the latest publications and public databases, including GRASP and TCGA. We removed all the variants that occurred in the training set of PAFA from these testing sets. We used AUC values and *p* values (Wilcoxon rank-sum test) to evaluate the performance of these methods.

### Construction of the online platform

To facilitate the use of PAFA, we built an online platform for the navigation or batch download of target variants. This platform was developed using Java and was deployed on a Tomcat server. We developed the user interface using HTML5, JavaScript, and D3.js. In addition to conveniently accessing PAFA scores, the online platform incorporates other functions, such as evaluating target variants relying on prior databases containing disease-associated genes, providing enrichment analysis on variant set and relevant annotation information from 1000 Genomes and genomic databases, such as ENCODE and OMIM.

To evaluate variants using information from gene-disease databases (e.g., OMIM, GAD), we first mapped the variants to a range of annotated elements, such as exons, TSS, 3′-UTR, 5′-UTR, enhancers, TFBS, and open chromatin, based on the abundant annotation source integrated in our database. As variants in different types of elements cause discrepant influences on gene expression, we set empirical weights to variants based on different types of elements. In addition, the proportion of overlapping section was considered. After retrieving the involved genes, a quantitative value was assigned to the variants in the following way, according to the occurrence frequency of genes in current gene-disease databases.

Assume that the target variant overlapped with *m* different genes and *n* different elements were influenced by each gene. Then, let ${L}_{e_j}$be the *i*th gene’s *j*th element’s length and ${L}_{o_{ij}}$ be the overlapped length between variant and the *i*th gene’s *j*th element. *W*_*T*_ represents the weight value of the type of element, which is set to 1.0 for exon and TSS, 0.5 for 3′-UTR and 5′-UTR, 0.3 for enhancer, 0.2 for TFBS (PeakSeq) and TFBS (SPP) in the gene, and 0.1 for the gene’s open chromatin. *s*_*i*_ indicates the *i*th gene’s frequency as it appears in gene-disease databases. The score is calculated as

$$ score={\sum}_{i=1}^m{S}_i $$

with

$$ {S}_i={s}_i\times \mathit{\min}1,{\sum}_{j=1}^n\frac{L_{o_{ij}}}{L_{e_j}}\times {W}_T $$

To provide enrichment analysis for the target variant sets, we included background variant sets, such as variants from 1000 Genomes, genomic annotations from ENCODE, and canonical pathways in the Molecular Signatures Database (MSigDB). First, we mapped the test variants and background variants (user uploaded or selected) to a range of annotated elements. Then, we obtained genes related to the test and background variants. Next, we extracted the related pathways of these genes in MSigDB. Finally, according to the relationships among variants, genes, and pathways, we calculated the *p* value to estimate the enrichment degree in relevant pathways using Fisher’s exact test.

## Results

### Population differentiation of genetic variants associated with complex diseases or traits

**Fig. 2:**
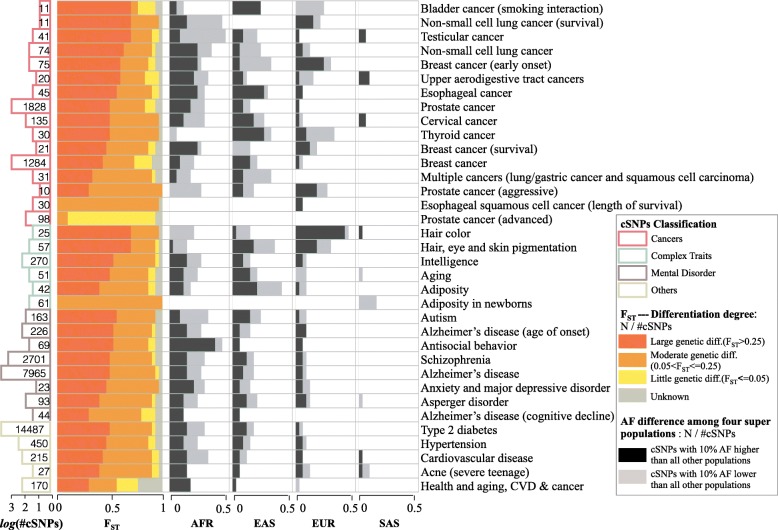
Population differentiation of genetic variants associated with complex diseases or traits. Thirty-five complex trait-/disease-associated SNP (cSNP) sets from GWASdb are listed. The color of the left-most bars represents the different types of diseases/traits, and the length of the bars represents the number of cSNPs in the sets. Adjacent stack bars represent the percentage of different differentiation degrees among each cSNPs set. The following four column stack bars exhibit different degrees of allele frequencies among super populations, namely, African (AFR), East Asian (EAS), European (EUR), and South Asian (SAS)

To explore the relationship between the population differentiation of genetic variants and common complex diseases or traits, we extracted SNPs associated with complex traits or diseases (cSNPs) from GWASdb [24]. Multiple categories of diseases/traits were chosen, including cancers, cardiovascular diseases, and mental disorders, as well as complex traits, such as hair color, adiposity, and intelligence (Fig. 2). Subsequently, we obtained population-specific allele frequencies of these cSNPs in four super populations (AFR, EAS, EUR, and SAS) derived from the 1000 Genomes project (Phase 3 variant calls). The corresponding allele frequency spectra in super populations for each disease/trait category were visualized in violin graphs. As shown in Additional file 1: Figure S9–12, cSNPs associated with different diseases or traits exhibited noticeably different allele frequencies, with certain diseases overrepresented in specific populations. For example, over 50% of testicular cancer-related SNPs occurred in more than half of Europeans, but in less than 25% of Africans. This is consistent with epidemiologic findings that testicular cancer incidence consistently remained the highest in Northern European populations and the lowest in African populations [53, 54]. To investigate the population genetic predispositions underlying these cSNPs, we introduced the fixation index (*F*_ST_), which is a measure of population differentiation due to genetic structure. With allele frequencies and sample sizes of the five super populations available from 1000 Genomes, we calculated the unbiased estimates of *F*_ST_ for cSNPs. The frequency distribution of *F*_ST_ for each disease/trait category was shown in Additional file 1: Figure S13. According to the criteria [55] that *F*_ST_ lower than 0.05 means little genetic differentiation and *F*_ST_ larger than 0.25 means high genetic differentiation, most of the cSNPs exhibited high genetic differentiations in human populations.

Furthermore, we examined 35 cSNP sets to get a comprehensive look at the relationship between the population differentiation of cSNPs and their associated complex diseases/traits. As shown in Fig. 2, the proportion of cSNPs with large (*F*_ST_ > 0.25), moderate (0.05 < *F*_ST_ ≤ 0.25), and little (F_ST_ ≤ 0.05) genetic differentiation was displayed in different colors. The number of cSNPs with 10% allele frequencies that were higher or lower than all other populations was calculated in each category based on known allele frequencies in human populations. As illustrated in the four right-most column stack bars of Fig. 2, the dark gray bar represents the frequency of cSNPs in a super population that is higher than all other super populations, and the light gray bar represents the frequency of cSNPs in a super population that is lower than all other super populations. Clearly, the majority of cSNPs display a strong preference towards specific human populations. For example, cSNPs associated with hair color showed a high occurrence in the European population, with nearly a half of them frequently occurring in European populations, which is consistent with previous studies [56, 57]. More examples can be seen in cancer-related diseases. Of the 1828 prostate cancer-related SNPs curated from multiple literature sources, more than half showed great genetic differentiation among super populations. Out of these cSNPs, 720 were reported to likely occur in the African population, but not in other populations. In fact, African American men have the highest prostate cancer incidence rate in the world, although the rate in the African population is unclear [58, 59]. Taken together, these examples indicate that different cSNPs exhibit various levels of population differentiation, and the incorporation of *F*_ST_ or other allele frequency features may help evaluate the significance of human genetic variants.

### The PAFA approach

**Fig. 3:**
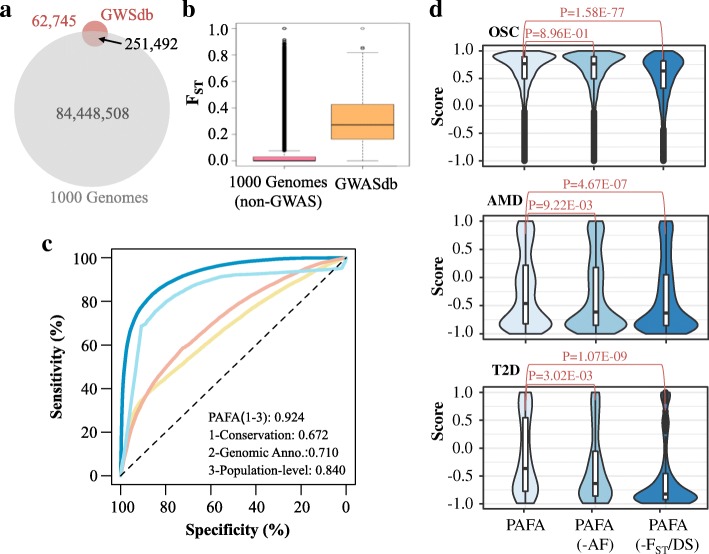
Evaluation of features used in PAFA. **a** A Venn diagram on variant sets from GWASdb and 1000 Genomes. The overlapping section represents the mutations that exist in both GWASdb and 1000 Genomes. **b** *F*_ST_ scores for cSNPs in GWASdb versus variants in 1000 Genomes after removing variants in GWASdb. **c** Tenfold cross-validations are applied to evaluate the performance of PAFA and three PAFA classifiers that were separately constructed on the three feature groups. Receiver operating characteristic (ROC) curves are exhibited. The dashed line indicates random chance. The value of the area under the curve (AUC) is calculated for each feature. **d** Violin plots of PAFA scores for three disease-related variant sets, including ovarian serous cystadenocarcinoma (OSC), age-related macular degeneration (AMD), and type 2 diabetes (T2D). PAFA scores are provided by the classifier that was constructed based on all features (PAFA), all except allele frequencies (-AF) and all except *F*_ST_ and dispersion score (-F_ST_/DS). The *p* values for comparisons of these scores were calculated by Wilcoxon rank-sum test

The PAFA algorithm contains two components: prioritizing functional genetic variants and annotating variants by integrating a priori functional genomic data (Fig. 1). To discriminate potential functional variants from background variants, sparse logistic regression with L1 regularization was applied to train a noncoding sensitive discriminative model. To be noncoding sensitive, PAFA utilizes training data sets located in noncoding regions and selects features with the ability to prioritize noncoding variants. First, the training variants were partly derived from ClinVar, GWASdb v2, and 1000 Genomes. PAFA classified them into two distinct variant sets (functional and control) with multiple filtration steps, including filtering duplicates and conflicting records, selecting element-overlapping SNPs associated with complex traits or diseases, and measuring the similarity between common variants from 1000 Genomes and functional variants based on various annotations. For the functional variant set, variants annotated as “pathogenic” in ClinVar were first selected; these variants are mainly located in coding regions. Considering that genome-wide association studies have reliably linked coding or noncoding genetic variants to complex diseases or traits, significant cSNPs (*p* value < 10E−8) in GWASdb were selected as another source of the functional variant set. Noncoding SNPs may affect target genes by disrupting their normal regulatory mechanism. To reduce the number of selected noncoding variants, we only chose the variants that overlapped with any genomic elements from ENCODE [7] and UTRdb [29]. For the control variant set, variants annotated as “benign” in ClinVar were the first data source. Common variants from 1000 Genomes were usually labeled benign by previous supervised classifiers (e.g., GWAVA [17]). However, more than 80% of curated SNPs archived in GWASdb are also present in the SNP list of 1000 Genomes, since most GWAS studies have been performed using genotyping SNP arrays based on common variants (Fig. 3a). In addition to these shared SNPs in GWASdb and 1000 Genomes, a large number of unannotated common variants in 1000 Genomes may also be clinically important, as the number of diseases and traits studied by GWAS is still not sufficiently comprehensive [60]. Ignoring or misclassifying these potential functional SNPs may lead to an incorrect assessment of target genetic variants. Therefore, we employed the *F*_ST_ index as a metric to infer the potential biological significance of variants in 1000 Genomes. We first compared the distribution of *F*_ST_ indexes for SNPs in 1000 Genomes with those in GWASdb, and we found that over 90% of variants in 1000 Genomes showed little genetic differentiation (Fig. 3b). In contrast, more than half of the cSNPs in GWASdb displayed enhanced genetic differentiation. A considerable fraction of variants (over 6 million out of 84 million) show high population differentiation indices (> 0.25) in 1000 Genomes, the number of which is much larger than the total number of variants presented in GWASdb. To generate the control variant set, we first constructed a regressive model based on various features of the cSNPs used in the training stage. Then, we selected variants with very low *F*_ST_ values (< 0.01) from 1000 Genomes. The constructed regressive model was used to rank the selected low-*F*_ST_ variants. According to the rank of variants, we selected the variants that were inversely associated with cSNPs utilized by PAFA as another source for the control variant set. Finally, PAFA incorporated simulated rare benign variants as part of the control variant set.

The features used in PAFA can be classified into three categories: population-level metrics, evolutionary conservation, and genomic annotations. These features show distinct patterns in the functional and control variant sets and exhibit certain abilities in annotating noncoding variants. Among these features, population-level metrics consist of fixation index (*F*_ST_), allele frequencies, and dispersion score (DS) that is calculated based on allele frequencies. *F*_ST_, which is frequently used as a summary of genetic differentiation among groups, was first introduced as a feature to distinguish between functional and background variants. DS was also incorporated, as standard deviation is by far the most widely used measure of dispersion. As shown in Fig. 3c, the population-level features alone exhibited better performance than all other feature groups, with an AUC of 0.840 (tenfold cross-validation). To reveal which feature has a stronger impact in the PAFA model, we selected three variants sets related to complex diseases, including 6147 variants related to ovarian serous cystadenocarcinoma (OSC) from the TCGA database [34], 1339 variants associated with age-related macular degeneration in East Asians [61], and 150 variants related to type 2 diabetes from European populations [62]. These diseases are all reported to be population-relevant [63–66]. As shown in Fig. 3d, the constructed model based on the combination of *F*_ST_, DS, and allele frequencies exhibited much better performance in evaluating these variants associated with complex diseases than the model based on allele frequencies or *F*_ST_ alone. In addition, the PAFA classifiers without features of *F*_ST_ and DS exhibited a poorer performance than the classifiers without allele frequencies, according to the *p* values calculated by a Wilcoxon rank-sum test (Fig. 3d).

In addition to prioritizing functional variants, PAFA also provides gene-centric annotations for both coding and noncoding variants. PAFA integrates genetic elements, including annotated enhancer, TSS, exon, 3′-UTR, and 5′-UTR, from curated databases, including ENCODE. To test the reliability of the annotated genomic elements, functional enrichment analysis was performed on 10,143 de novo mutations identified from 200 autism spectrum disorder (ASD) parent-child trios [67]. Among these mutations, 5635 variants do not have any overlap with annotated coding regions. We obtained the affected genes of coding and noncoding variants through the gene-centric database of PAFA, and we performed enrichment analysis on these genes separately using DAVID [68]. All enriched pathways listed in Additional file 1: Figure S14 were reported to be closely related to ASD in previous studies. Several pathways were apparently enriched in the affected gene sets of both coding and noncoding variants, indicating that these noncoding variants may cooperate with coding variants in the development of ASD. For example, the affected gene sets of both coding and noncoding variants were enriched in the cAMP pathway, with Benjamini-adjusted *p* values of 3.31E−05 and 5.81E−05, respectively. The affected gene sets of coding and noncoding variants are not consistent, with several enriched pathways that are specific to noncoding variants (Additional file 1: Figure S14).

### Performance comparison on prioritizing functional variants

To evaluate the performance of PAFA on prioritizing functional variants from background variants, we compared PAFA with seven widely used prioritization methods, CADD [12], FATHMM-MKL [13], DANN [16], GWAVA [17], DIVAN [18], Eigen [11], and LINSIGHT [19]. A detailed comparison of these tools is shown in Additional file 1: Table S1. Considering that FATHMM-MKL provides two different scores by using different features, namely, a “coding score” and a “noncoding score,” PAFA was compared with both of them. Eigen provides Eigen and Eigen-PC scores by using different algorithm strategies, and GWAVA provides Region, TSS, and Unmatched scores by using different training datasets; thus, all these scores were included in the comparison. Similarly, the Region and TSS scores provided by DIVAN were also included. For comparison, we downloaded pre-computed GWAVA (v1.0), DANN (Oct 10, 2014), DIVAN (Dec 6, 2016), Eigen (Jan 4, 2016), and LINSIGHT (Aug 15, 2016) scores from their source websites, and we obtained CADD (v1.3) and FATHMM-MKL (Jan 11, 2015) scores through their online retrieval systems.

**Fig. 4:**
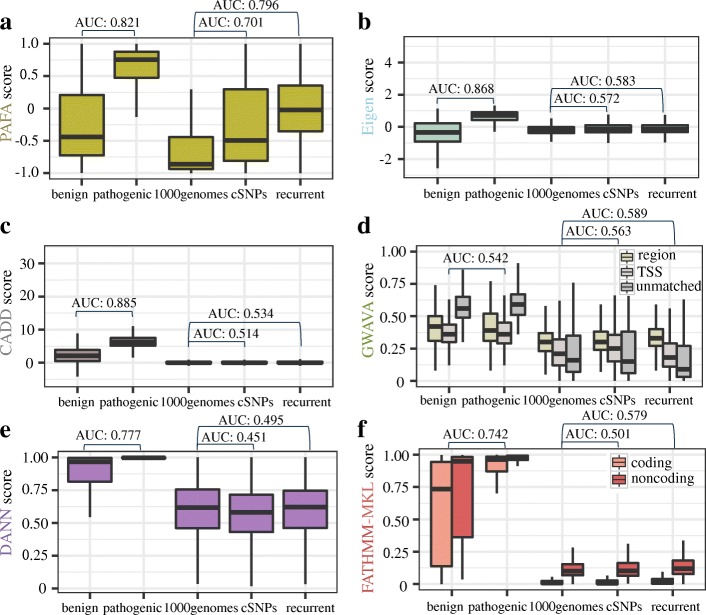
Performance comparison on prioritizing functional variants. Distribution of scores for evaluating five different variant sets, from left to right: variants labeled “benign/likely benign” in ClinVar, variants labeled “pathogenic/likely pathogenic” in ClinVar, randomly selected variants from 1000 Genomes, significant trait-/disease-associated SNPs (cSNPs) from GWAS Catalog and recurrent cancer-related mutations in noncoding regions from COSMIC. **a** Boxplot for PAFA scores. **b** Boxplot for Eigen scores. **c** Boxplot for *C* scores of CADD v1.3. **d** Boxplot for Region, TSS, and Unmatched scores of GWAVA. **e** Boxplot for DANN scores. **f** Boxplot for coding and noncoding scores of FATHMM-MKL. Area under the curve (AUC) values were calculated for each tool to evaluate their performance on (1) prioritizing pathogenic variants from benign ones, most of which are located in coding regions; (2) prioritizing cSNPs from common variants in 1000 Genomes; and (3) prioritizing noncoding recurrent variants from common variants in 1000 Genomes. As GWAVA and FATHMM-MKL provide more than one score, we display the AUC value with the best performance

We first performed a comprehensive evaluation of seven tools (PAFA, Eigen, CADD, GWAVA, DANN, FATHMM-MKL, and LINSIGHT) by assessing five different types of variant sets, including benign and pathogenic coding variants, common variants with frequencies of at least 1% in the populations studied in 1000 Genomes, cSNPs, and recurrent noncoding variants (Fig. 4). LINSIGHT was not involved in assessing pathogenic and benign coding variants because it mainly inferred the selective pressure on noncoding sites and only provided scores for noncoding sites. We randomly selected 12,035 variants in chromosome 22 from the 1000 Genomes project (Phase 3 variant calls), and we downloaded the variant data sets used in the Eigen paper, which presented the four other types of variant data sets. In total, we obtained 16,545 pathogenic/likely pathogenic variants and 3482 benign or likely benign nonsynonymous variants from the ClinVar database, 121,507 recurrent somatic noncoding variants in the COSMIC database [1], and 14,915 cSNPs that were found to be genome-wide significant and were reported in the GWAS Catalog at the National Human Genome Research Institute (NHGRI). After removing the variants used in the training dataset, the common variants were reduced to a total of 11,035 variants, and the benign/likely benign variants and pathogenic/likely pathogenic variants were reduced to 1671 and 2429, respectively. Similarly, the number of recurrent variants and cSNPs from the GWAS Catalog were reduced to 120,437 and 11,570, respectively. After generating these five datasets, PAFA and the six other tools were used to compute scores to evaluate these variants.

As shown in Additional file 1: Table S2, all tested tools (except for GWAVA, which aims to predict the functional impact of noncoding genetic variants) perform well in distinguishing pathogenic coding variants from benign ones, with AUC values ranging from 0.702 to 0.885. However, when we evaluated their abilities in prioritizing recurrent noncoding variants and cSNPs from common variants in the 1000 Genomes, some tools exhibited decreased performance with AUC values < 0.5 (Fig. 4e and Additional file 1: Table S2). Besides PAFA, which performed well with an AUC value of 0.701 (Fig. 4a), Region score of GWAVA, Eigen, coding, and noncoding scores of FATHMM-MKL and CADD exhibited moderate performance in distinguishing recurrent noncoding variants from common variants with AUC values ranging from 0.534 to 0.589 (Fig. 4 b–d, f). In general, among these supervised and unsupervised algorithms, PAFA achieved comparable performance to CADD and Eigen on pathogenic coding variants, with an AUC value of 0.821. Remarkably, PAFA exhibited the best performance in discriminating multiple types of variants associated with complex diseases or traits, with an AUC value of 0.796 for prioritizing recurrent noncoding variants and an AUC value of 0.701 for prioritizing cSNPs (Fig. 4a and Additional file 1: Table S2).

### Prioritizing coding risk variants

We first compared the performance of PAFA, Eigen, CADD, GWAVA, FATHMM-MKL, and DANN on prioritizing coding variants from five well-studied genes (MLL2, CFTR, BRCA1, BRCA2, and TERT) associated with Kabuki syndrome, cystic fibrosis, breast cancer, or aggressive thyroid tumor, respectively. After removing variants used in the training stage of PAFA, we obtained 37 disease-associated variants in BRCA1, 15 in BRCA2, 41 in CFTR, 92 in MLL2, and 42 in TERT. At the same time, variants of these genes that were labeled benign in ClinVar were obtained for comparison. The *p* values were determined by comparing the scores of disease-associated variants with those of benign variants using a Wilcoxon rank-sum test. As shown in Additional file 1: Table S3, for variants in BRCA1, CFTR, and MLL2, most tools could identify risk variants from benign ones with *p* values smaller than 0.05. However, for variants in BRCA2 and TERT, PAFA outperformed all other methods by prioritizing more variants with much more significant *p* values.

We further evaluated PAFA’s performance in prioritizing risk coding variants from adjacent variants utilizing two cancer-related coding variant sets. The first dataset includes 916 driver variants that were identified from 560 breast cancer samples in a recent study [37]. These driver variants are all located in coding regions. Correspondingly, variants adjacent to these disease-related variants (50 bp upstream and downstream) were extracted from 1000 Genomes and were used as the control dataset. As shown in Additional file 1: Figure S15A, previous methods, such as CADD, DANN, and FATHMM-MKL, exhibited good performance on prioritizing rare pathogenic variants from adjacent common variants, with AUC values larger than 0.8. PAFA exhibited the best performance with an AUC value of 0.947. Without population-level metrics, the PAFA classifier exhibited a decreased AUC value dropping to 0.714 (Additional file 1: Figure S16A–16B), which indicates that population-level metrics can greatly improve the performance of PAFA in discriminating coding risk variants from common variants. To further explore the role of various features used in the PAFA classifier, we constructed three models using different features. As shown in Additional file 1: Figure S16C–16E, all three features, including evolutionary conservation, genomic annotations, and population-level metrics, exhibited the ability to prioritize these breast cancer-related variants. Among them, population-level metrics showed an AUC value of 0.969, where common variants with low population differentiation indexes were assigned relatively low scores, and rare variants exhibited an even distribution of scores. In the following, we constructed five different models using various combinations of the training datasets (Additional file 1: Figure S16F–16J). After removing pathogenic rare variants from the training datasets, the PAFA classifier cannot distinguish breast cancer-related rare variants from adjacent common variants, with an AUC value of 0.052 (Additional file 1: Figure S16F).

The second dataset contains 6133 ovarian serous cystadenocarcinoma (OSC)-related variants that were obtained from the TCGA database [69]. More than 97% of these variants overlapped with exons. Correspondingly, we generated rare noncoding variants as the control dataset by simulating variants of the number of OSC-associated variants ten times; these variants are adjacent to the risk rare variants (50 bp upstream and downstream). PAFA, CADD, FATHMM-MKL, and LINSIGHT exhibited an ability to discriminate OSC-associated variants from simulated rare noncoding variants, with AUC values ranging from 0.565 to 0.682 (Additional file 1: Figure S15B), and PAFA exhibited the best performance. We constructed different PAFA classifiers to test feature groups as described above (Additional file 1: Figure S17A–E). The PAFA classifier that was constructed based on genomic annotations exhibited fairly good performance, with an AUC value of 0.747 (Additional file 1: Figure S17C), and the classifier constructed based on evolutionary conservation features had a moderate performance, with an AUC value of 0.543 (Additional file 1: Figure S17D). As expected, when the PAFA classifier utilized population-level metrics alone, it had no ability in discriminating risk rare variants from simulated ones, with an AUC value of 0.48 (Additional file 1: Figure S17E), since we only had population differentiation information of variants with frequencies of at least 1% in the populations studied in the 1000 Genomes. However, PAFA performed better than the PAFA classifier without population-level features (Additional file 1: Figure S17A–B). In addition, we found that the pathogenic rare variants and simulated rare benign noncoding variants in the training datasets also contributed to the performance of PAFA (Additional file 1: Figure S17F–17J).

### Applying PAFA to noncoding genetic variants associated with complex diseases

**Fig. 5:**
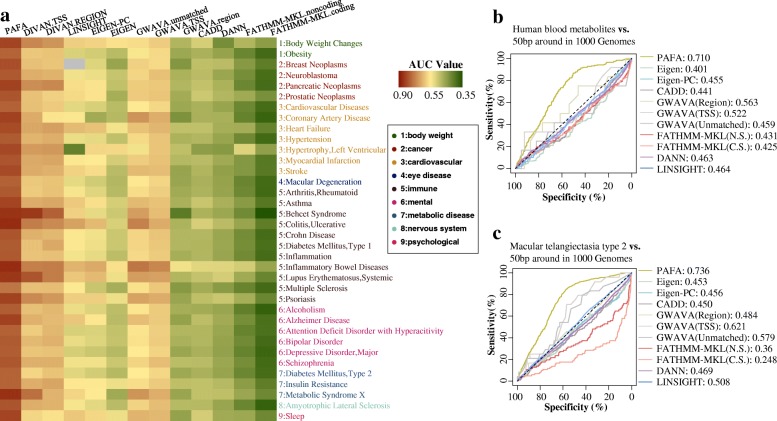
Performance comparison on prioritizing noncoding variants associated with complex diseases. **a** Heatmap of AUC values for performance comparison among PAFA, DIVAN, LINSIGHT, Eigen, GWAVA, CADD, DANN, and FATHMM-MKL across 36 investigated diseases. **b** Prioritization of 221 variants associated with human blood metabolites identified by genome-wide analyses from 706 adjacent variants extracted from 1000 Genomes. **c** Prioritization of 647 common variants associated with macular telangiectasia type 2 from 1764 adjacent variants extracted from 1000 Genomes

To assess PAFA’s performance in prioritizing noncoding variants associated with complex diseases and traits, we first compared PAFA with seven tools, namely, DIVAN, LINSIGHT, Eigen, GWAVA, CADD, DANN, and FATHMM-MKL. The tools were used to prioritize diseases or traits related to variants in the GRASP database [35], which includes approximately 8.87 million SNPs identified from 2082 GWAS. Considering that DIVAN constructed a specific classifier for each disease and only provided scores for variants related to 45 diseases, we selected 36 matched variant sets in GRASP as a test dataset; over 85% of these variants were noncoding common variants. The corresponding benign variants were randomly selected by sampling all the risk variants from the 1000 Genomes ten times; all GRASP variants were excluded from sampling. As shown in Fig. 5a, CADD, DANN, FATHMM-MKL, and the Region scores provided by GWAVA exhibited poor ability in prioritizing common variants associated with complex diseases/traits. Unsupervised methods, namely, LINSIGHT and Eigen, exhibited moderate performance in some diseases/traits, such as heart failure and ulcerative colitis, with AUC values larger than 0.55. PAFA achieved the best performance out of all methods, with AUC values in the range of 0.738–0.858 (median 0.799), followed by the performance of TSS and Region scores provided by DIVAN and Unmatched and TSS scores provided by GWAVA. DIVAN achieved comparable performance with PAFA in several immune diseases, such as Behcet syndrome and systemic lupus erythematosus. Moreover, we found that PAFA outperformed all other methods in prioritizing variants associated with immune-related diseases, cancers, and cardiovascular diseases, with a median AUC value of 0.810.

Next, we selected two of the most recently published functional variant sets identified by genome-wide association studies to further evaluate the performance of PAFA. The first dataset includes 221 variants associated with human blood metabolites [38], among which 150 are common noncoding variants. The second dataset contains 647 common variants associated with macular telangiectasia type 2 [39], among which are 554 common noncoding variants. For each dataset, variants adjacent to the disease-related variants were extracted from 1000 Genomes and were used as the corresponding control dataset. Ultimately, we obtained 706 variants for human blood metabolites and 1764 for macular telangiectasia type 2 as control datasets. As shown in Fig. 5b, c, PAFA exhibited the best performance out of all the tools in prioritizing these complex disease/traits related to variants identified by whole-genome sequencing, with an AUC of 0.710 for human blood metabolites and 0.736 for macular telangiectasia type 2. The Region scores provided by GWAVA exhibited moderate performance in prioritizing blood metabolites, with an AUC of 0.563. The TSS and Unmatched scores provided by GWAVA exhibited an ability to prioritize macular telangiectasia type 2, with AUC values of 0.621 and 0.579, respectively. Except for PAFA and GWAVA, the other tools exhibited a poor ability to prioritize the two functional variant datasets identified from GWAS. Genomic annotations and conservation metrics were not efficient to discriminate common noncoding functional variants from common noncoding neutral variants (Additional file 1: Figure S18A–18B, S19A–19B). For blood metabolites and macular telangiectasia type 2, the PAFA classifier constructed based on population-level metrics had improved performance, with AUC values of 0.884 and 0.826, respectively (Additional file 1: Figure S18C, S19C). In addition, the training data from GWASdb and 1000 Genomes also contributed to the performance of PAFA (Additional file 1: Figure S18F–18G, S19F–19G). Without the training data from GWASdb or 1000 Genomes, the PAFA classifier exhibited a dramatic decrease in its ability to prioritize common variants associated with diseases from adjacent common variants, with AUC values of 0.405 and 0.556, respectively, for human blood metabolite-related variants and AUC values of 0.350 and 0.559, respectively, for macular telangiectasia type 2-related variants.

### Applying PAFA to cancer-related noncoding variants

**Fig. 6:**
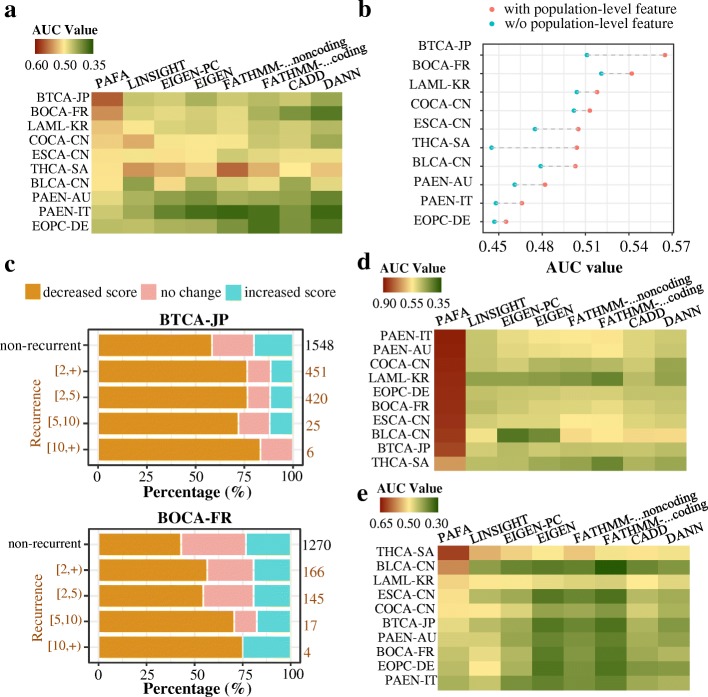
Performance evaluation on cancer-related noncoding risk variants. **a** Common recurrent noncoding variants versus common non-recurrent noncoding variants. **b** Prioritizing recurrent variants from non-recurrent variants using PAFA with or without population-level features across ten cancer-related variant sets from ICGC. **c** Alteration of PAFA scores after removing population-level features in BTCA-JP and BOCA-FR. **d** Rare noncoding variants versus adjacent common noncoding variants extracted from 1000 Genomes. **e** Rare recurrent noncoding variants versus rare non-recurrent noncoding variants

To assess PAFA’s ability in discriminating common recurrent from non-recurrent variants, we selected ten cancer-related variant sets from ICGC Cancer Genome Projects [36] (Additional file 1: Table S4). We selected variants located in noncoding regions as well as those recorded in 1000 Genomes, obtaining 127~74,685 noncoding common variants for each cancer dataset. Here, we deemed a noncoding variant that was observed in at least two donors of a specific cancer as a recurrent noncoding variant. As shown in Fig. 6a, for common variants from THCA-SA, most of the methods exhibited an ability to prioritize recurrent variants from non-recurrent ones, with AUC values ranging from 0.5 to 0.56. However, except for PAFA, these methods exhibited poor performance for variants from other projects. For variants from BTCA-JP, BOCA-FR, LAML-KR, PAEN-AU, PAEN-IT, and EOPC-DE, most of the previous methods (LINSIGHT, Eigen, FATHMM-MKL, CADD, and DANN) could not discriminate recurrent from non-recurrent noncoding variants, with AUC values ranging from 0.369 to 0.496. PAFA, however, exhibited better performance in prioritizing recurrent noncoding variants, with AUC values larger than 0.5 for seven out of ten samples.

To explore the role of population-level metrics on common noncoding variants introduced in the PAFA classifier, we tested its ability in prioritizing recurrent variants from non-recurrent ones by removing population-level features. Under default settings, the PAFA classifier exhibited better performance in discriminating common recurrent variants according to ten cancer-related variant sets from ICGC (Fig. 6b). After removing population-level features, however, recurrent variants tended to have more decreased scores than non-recurrent variants (Fig. 6c).

To assess PAFA’s ability in discriminating rare recurrent variants, we first compared PAFA’s performance in prioritizing noncoding rare risk variants from adjacent common variants with other algorithms. For variants from ICGC, we selected variants located in noncoding regions as well as those not recorded in 1000 Genomes, and we obtained 963, 242,564, 5408, 129,975, 31,632, 76,763, 95,741, 25,388, 1181, and 111,088 noncoding rare variants for BLCA-CN, COCA-CN, ESCA-CN, PAEN-AU, BOCA-FR, EOPC-DE, PAEN-IT, BTCA-JP, THCA-SA, and LAML-KR, respectively (Additional file 1: Table S4). For each of the ten variant sets from ICGC, benign variants adjacent to the cancer-related variants (50 bp upstream and downstream) were extracted from 1000 Genomes and were used as control datasets. As shown in Fig. 6d, the previous methods exhibited unstable and relatively poor performance in discriminating noncoding rare risk variants from adjacent common variants. For example, CADD, DANN, Eigen, and FATHMM-MKL exhibited an ability to prioritize rare risk variants from PAEN-IT, PAEN-AU, BOCA-FR, and ESCA-CN, with AUC values ranging from 0.502 to 0.561, but they could not discriminate cancer-related variants from LAML-KR and THCA-SA, with AUC values ranging from 0.414 to 0.488. PAFA outperformed all other methods in all ten variant sets, with AUC values ranging from 0.682 to 0.877. As shown in Additional file 1: Figure S20, the good performance of PAFA relied on the introduction of population differentiation features. Because these benign common variants are close to the cancer-related variants, prioritization methods that were solely based on genomic annotations did not perform well. According to LINSIGHT scores, noncoding rare risk variants from ESCA-CN, BLCA-CN, and BTCA-JP exhibited higher degrees of evolutionary constraint than adjacent common variants, with AUC values ranging from 0.509 to 0.539, but the noncoding rare risk variants from the other seven projects exhibited indistinguishable or lower degrees of evolutionary constraint compared with adjacent common variants, with AUC values ranging from 0.452 to 0.5. Moreover, we assessed the six tools’ performance in prioritizing rare recurrent noncoding variants, which are population-irrelevant. As shown in Fig. 6e, all current methods exhibited poor performance in prioritizing rare recurrent noncoding variants from non-recurrent ones. Among these algorithms, PAFA achieved the best performance in THCA-SA, BLCA-CN, LAML-KR, ESCA-CN, COCA-CN, BTCA-JP, and PAEN-AU. LINSIGHT, CADD, DANN, Eigen, and FAHMM-MKL exhibited an ability to discriminate rare recurrent noncoding variants in THCA-SA, with AUC values larger than 0.5. LINSIGHT also exhibited a slight discrimination ability for variants from BLCA-CN, COCA-CN, and EOPC-DE, with AUC values of 0.503, 0.503, and 0.51, respectively.

### An integrated online platform for PAFA

We developed an online platform to facilitate the use of PAFA. Precomputed PAFA scores for all 231 million variants of dbSNP and 2.68 billion single nucleotide variants throughout the human genome are integrated into this online platform, where users can access these precomputed PAFA scores through batch download or can submit a list of genomic locations or variants of interest to obtain target PAFA scores and gene-centric annotations. As shown in Additional file 1: Figure S6, this platform provides a simple and intuitive interface to help users determine the functional significance of variants. Its major functions include performing enrichment analysis for variants; annotating variants with known risk variants, genomic annotations, disease-associated genes, and pathways recorded in curated databases (e.g., ClinVar, COSMIC, and ENCODE); and providing scores for target variants based on their associated genes’ occurrence frequency in disease-related databases. Moreover, for variants that are found in 1000 Genomes, population-level information is also shown, including the *F*_ST_ index, allele frequency spectrum, and associated genomic elements. All curated knowledge data have been stored in MySQL, the platform was developed in JAVA, and the interactive interface was created with HTML5 and JavaScript.

## Discussion

PAFA is an integrative method for prioritizing clinically relevant variants from background variants by concentrating on factors associated with population-specific diseases/traits and common variants with weak effect. Through extensive evaluations, we demonstrated that PAFA consistently outperforms the latest published supervised and unsupervised methods. First, PAFA exhibits the best performance in discriminating significant cSNPs in the GWAS Catalog and recurrent somatic noncoding variants in the COSMIC database from common variants in 1000 Genomes. Second, PAFA exhibits high sensitivity and specificity in prioritizing pathogenic variants related to Mendelian diseases as well as prioritizing coding risk variants from both adjacent common variants and simulated noncoding variants. Third, PAFA has an increased capability to prioritize common variants related to 36 complex diseases/traits and two newly published variant sets identified by genome-wide analyses from background variants with population-level metrics. It also outperforms previous methods in discriminating recurrent common noncoding variants from non-recurrent noncoding variants. In addition, PAFA also provides gene-centric functional annotations for variants based on the integrated annotated functional elements. To facilitate its usage, we developed a user-friendly online platform that not only allows users to evaluate variant sets by PAFA but also provides multiple functions, including enrichment analysis on target variant sets and comprehensive annotations for variants/genes.

Previous supervised classifiers are good at prioritizing coding functional variants but have limited ability to prioritize and annotate noncoding variants. As an unsupervised method, Eigen avoids the problem caused by erroneous labeling, but it still cannot distinguish complex disease-/trait-associated variants from the background variants. PAFA exhibited much better performance in prioritizing noncoding variants than the currently available approaches, no matter supervised or unsupervised methods, under comprehensive evaluations. Its good performance can be attributed to three types of improvements. First, PAFA employs a population differentiation index to prioritize population-specific associations of common risk variants for complex diseases. This feature relies on the variants in Phase 3 of 1000 Genomes and considers both population difference and diversity within populations by calculating *F*_ST_ and dispersion score (DS). It improves the performance of PAFA in prioritizing common functional noncoding variants from background variants. Second, the optimization of training datasets from GWASdb and 1000 Genomes helps PAFA prioritize common functional variants and reduce its false-positive rate. Due to the limited amount of verified functional noncoding variants, we introduced cSNPs from GWAS, which is composed of tens of thousands of loci associated with various human diseases and traits. Since noncoding variants are suspected of disrupting the normal regulatory control mechanisms of target genes [18], we selected significant cSNPs that overlap genomic elements from GWASdb as functional variants in the training stage. Considering that 1000 Genomes may contain a large number of functionally or clinically important variants, we selected likely benign variants from 1000 Genomes utilizing multiple filtration strategies. Based on the cSNPs used in the training stage, we constructed a regressive model and selected variants from 1000 Genomes with the least similarity and low population differentiation indices as control variants. Third, we integrated multiple genomic annotations and selected verified pathogenic variants from ClinVar as a part of PAFA’s training set, and the results showed that PAFA exhibited improved performance in prioritizing recurrent from non-recurrent variants and performed well in discriminating noncoding risk variants.

In this work, we describe PAFA, an effective method for the prioritization and functional assessment of genetic variants associated with complex traits or diseases, particularly population-relevant noncoding variants. PAFA employs a sophisticated model for feature integration by combining multiple features, including genomic annotations, evolutionary conservation metrics, and population differentiation metrics. The population differentiation index is adopted to improve predictive performance due to the high genetic differentiation of cSNPs among human populations. Genomic annotations help PAFA discriminate risk variants by the information of genomic elements the variants overlapped. Functional regions are more likely to have higher evolutionary conservation degree than neutral regions. These features work together by employing a sparse logistic regression algorithm with L1 regularization, and this algorithm is fit for sparse matrices by ignoring missing values. In addition, to avoid strong model assumptions, PAFA employs an integrative supervised approach. Considering that the number of verified noncoding variants is limited, PAFA selects the training set from curated databases with multiple filtration strategies. This combination of more efficient features and reliable training sets makes PAFA more powerful and robust than existing state-of-the-art methods, both supervised and unsupervised, in detecting functional noncoding variants. However, as a correctly labeled training set is the key to improving the sensitivity and accuracy of prioritization methods, more verified annotated noncoding variants are still expected. Pre-computed PAFA pathogenicity scores for 2.68 billion human SNVs based on the GRCh37/hg19 assembly are now available for batch download through our constructed online platform. Users can navigate variants based on either GRCh37/hg19 or GRCh38 using the lift-over tool integrated in PAFA. We will update the PAFA tool and its web portal when new versions of annotated noncoding variants and genomic annotations (e.g., ClinVar and ENCODE) are available. New released resources, like gnomAD frequency data [70], are expected to be integrated in a future update. We believe that PAFA will be an indispensable tool for prioritizing and annotating functional noncoding variants that are associated with complex traits or diseases.

## Conclusion

This study presents a novel supervised algorithm for prioritization and functional assessment of genetic variants associated with complex diseases or traits, especially for noncoding variants. It introduces measures to evaluate genetic differentiation of variants among different population groups. PAFA also recalibrates abundant training variants from curated databases with multiple new filtration strategies. Through comprehensive performance evaluations, as well as compared with previous methods, we demonstrated that PAFA exhibits a much better performance on prioritizing both coding and noncoding risk variants as well as discriminating recurrent from non-recurrent variants. We further constructed a user-friendly web server, which not only allows users to evaluate variants using PAFA but also provides comprehensive functional annotations by integrating abundant functional genomic elements.

### Availability and requirements

The availability and requirements are listed as follows:

Project name: PAFA

Project home page: http://159.226.67.237:8080/pafa; http://bioinfo.biols.ac.cn

Operating system(s): platform independent.

Programming language: Java, MySQL, JavaScript, HTML5.

Other requirements: Chrome, Firefox, Safari.

## Additional files

Additional file 1: **Table S1.** A tabular comparison between PAFA and seven other ensemble classifiers aimed at detecting functional/deleterious variants from background variants. **Table S2.** Comparisons among PAFA, Eigen, CADD, GWAVA, DANN, FATHMM-MKL, and LINSIGHT in evaluating variants from four curated databases, including ClinVar, 1000 Genomes, GWAS Catalog, and COSMIC. **Table S3.** Comparisons among PAFA, Eigen, CADD, GWAVA, FATHMM-MKL, and DANN in discriminating pathogenic variants from benign variants associated with Mendelian diseases. **Table S4.** Statistics often cancer-related variant sets from ICGC projects. **Figure S1.** Genetic and genomic resources used in PAFA and their screenshots. **Figure S2.** Genetic and genomic resources used in the 1000 GENOMES part of the PAFA online platform and their screenshots. **Figure S3.** Genetic and genomic resources used in the ANNOTATION part of the PAFA online platform and their screenshots. **Figure S4.** Genetic and genomic resources used in the VSEA part of the PAFA online platform and their screenshots. **Figure S5.** Genetic and genomic resources used in the SEARCH part of the PAFA online platform and their screenshots. **Figure S6.** An integrated PAFA online platform for variant prioritization and functional annotation. **Figure S7.** Flowchart of selecting and filtering training variants used in PAFA. **Figure S8.** Tenfold cross-validations are applied to evaluate the performance of features used in PAFA. **Figure S9.** Distribution of allele frequencies for 24 cancer-associated variant sets from GWASdb among super populations. **Figure S10.** Distribution of allele frequencies for nine complex trait-associated variant sets from GWASdb among super populations. **Figure S11.** Distribution of allele frequencies for eight mental disorder-associated variant sets. **Figure S12.** Distribution of allele frequencies for 17 complex disease-associated variant sets. **Figure S13.** Distribution of *F*_ST_ values for variant sets associated with complex diseases and traits. **Figure S14.** Enriched pathways of genes associated with coding and noncoding variants. **Figure S15.** Sensitivity and specificity of tools in distinguishing coding risk variants from adjacent variants. **Figure S16.** Distributions of PAFA scores for breast cancer-related variants and adjacent variants from the 1000 Genomes. **Figure S17.** Distributions of PAFA scores for OSC-related variants from TCGA and simulated noncoding rare variants. **Figure S18.** Distributions of PAFA scores for human blood metabolite-related variants and adjacent variants from 1000 Genomes. **Figure S19.** Distributions of PAFA scores for macular telangiectasia type 2-related variants and adjacent variants from 1000 Genomes. **Figure S20.** Distributions of PAFA scores for bladder cancer-related variants and adjacent variants from 1000 Genomes. (PDF 2069 kb)
