# Supplementary material for: Prioritization and functional assessment of noncoding variants associated with complex diseases
Source: Genome Med. 2018 Jul 11;10:53. doi: 10.1186/s13073-018-0565-y (PMC6042373; doi:10.1186/s13073-018-0565-y)
Supplement: Supplementary file 1 — Table S1. A tabular comparison between PAFA and seven other ensemble classifiers aimed at detecting functional/deleterious variants from background variants. Table S2. Comparisons among PAFA, Eigen, CADD, GWAVA, DANN, FATHMM-MKL, and LINSIGHT in evaluating variants from four curated databases, including ClinVar, 1000 Genomes, GWAS Catalog, and COSMIC. Table S3. Comparisons among PAFA, Eigen, CADD, GWAVA, FATHMM-MKL, and DANN in discriminating pathogenic variants from benign variants associated with Mendelian diseases. Table S4. Statistics often cancer-related variant sets from ICGC projects. Figure S1. Genetic and genomic resources used in PAFA and their screenshots. Figure S2. Genetic and genomic resources used in the 1000 GENOMES part of the PAFA online platform and their screenshots. Figure S3. Genetic and genomic resources used in the ANNOTATION part of the PAFA online platform and their screenshots. Figure S4. Genetic and genomic resources used in the VSEA part of the PAFA online platform and their screenshots. Figure S5. Genetic and genomic resources used in the SEARCH part of the PAFA online platform and their screenshots. Figure S6. An integrated PAFA online platform for variant prioritization and functional annotation. Figure S7. Flowchart of selecting and filtering training variants used in PAFA. Figure S8. Tenfold cross-validations are applied to evaluate the performance of features used in PAFA. Figure S9. Distribution of allele frequencies for 24 cancer-associated variant sets from GWASdb among super populations. Figure S10. Distribution of allele frequencies for nine complex trait-associated variant sets from GWASdb among super populations. Figure S11. Distribution of allele frequencies for eight mental disorder-associated variant sets. Figure S12. Distribution of allele frequencies for 17 complex disease-associated variant sets. Figure S13. Distribution of FST values for variant sets associated with complex diseases and traits. Figure S14. Enriched pa [file 13073_2018_565_MOESM1_ESM.pdf]

Table (S1-S4)

Table S1. A tabular comparison between PAFA and seven other ensemble classifiers aimed at detecting functional/deleterious variants from background variants.

	Type	Training set (functional/deleterious)	Training set (non-functional/benign)	Features
**PAFA**	Sparse logistic regression with L1 regularization	Variants annotated 'pathogenic' in ClinVar; SNP from GWASdb with p-value<10E-8 and genomic elements overlapped;	Variants annotated as 'Benign' in ClinVar; Variants in 1000 Genomes with low population differentiation	Evolutionary conservation annotations; Genomic annotations; Population differentiation indexes;
**CADD v1.3**	Logistic regression	Simulated de novo mutations	Variants in 1000 Genomes Project with DAF≥95%	All kinds of annotations could be found
**FATHMM-MKL**	Supervised SVM with a multiple kernel learning(MKL)	Heritable germ-line mutations in HGMD	SNVs in the 1000 Genomes Project	Evolutionary conservation annotations; Annotations from ENCODE; GC Content;
**DANN**	Deep neural network algorithm	Simulated de novo mutations	Human-Chimp fixed differences + 1000G above 95% derived	All kinds of annotations could be found
**GWAVA**	Supervised Random Forest	Variants annotated as 'regulatory mutations' in HGMD	Variants in 1000 Genomes Project (random selection/matched for distance to nearest TSS/ variants in the 1kb surrounding each of the HGMD variants)	Regulatory features; Genic context; Genome-wide properties;
**DIVAN**	Ensemble learning	Disease-specific SNPs in ARB	Variants in 1000 Genomes Project	Epigenomic annotation; Genomic annotation;
**Eigen**	Unsupervised spectral approach	/	/	Protein function scores/regulatory annotations; Evolutionary conservation annotations; Allele Frequencies;
**LINSIGHT**	Probabilistic evolutionary model	/	/	Conservation scores; Genomic annotation;

Table S2. Comparisons among PAFA, Eigen, CADD, GWAVA, DANN, FATHMM-MKL and LINSIGHT in evaluating variants from four curated databases, including ClinVar, 1000 Genomes, GWAS catalog and COSMIC. Area under the curve (AUC) values were calculated for each tool to evaluate their performance in 1) discriminating pathogenic coding variants from benign coding ones; 2) prioritizing noncoding recurrent variants from randomly selected common variants; 3) prioritizing cSNPs from randomly selected common variants.

**Tools**	**AUC values**		
	**Likely Pathogenic** vs. **Likely Benign** (ClinVar)	**1000genome** vs. **recurrent**	**1000genome** vs. **cSNP**
PAFA	0.821	0.796	0.701
Eigen	0.868	0.583	0.572
CADD v1.3	0.885	0.534	0.514
GWAVA.Region	0.494	0.589	0.518
GWAVA.TSS	0.487	0.457	0.563
GWAVA.Unmatched	0.542	0.396	0.493
DANN	0.777	0.495	0.451
FATHMM-MKL.Coding	0.742	0.579	0.458
FATHMM-MKL.Non-Coding	0.702	0.574	0.501
LINSIGHT	/	0.416	0.448

Table S3. Comparisons among PAFA, Eigen, CADD, GWAVA, FATHMM-MKL and DANN in discriminating pathogenic variants from benign variants associated with Mendelian diseases. Five genes, namely, BRCA1, BRCA2, CFTR, MLL2 and TERT were selected. Variants associated with BRCA1, BRCA2, CFTR, MLL2 were obtained from the Eigen website (Variants associated with BRCA1, BRCA2, CFTR, MLL2 were also treated as test sets in Eigen.); variants associated with TERT were obtained from the ClinVar website. We removed all the variants that occurred in the training set of PAFA. P values (Wilcoxon rank-sum test) were calculated for each tool.

Gene	n	Score	P value
BRCA1	37	PAFA	**1.04E-08**
		Eigen	3.32E-07
		CADD v1.3	8.07E-07
		GWAVA (Region Score)	3.42E-04
		GWAVA (TSS Score)	1.45E-03
		GWAVA (Unmatched Score)	2.16E-01
		FATHMM-MKL(Non-Coding Score)	2.64E-01
		FATHMM-MKL(Coding Score)	2.11E-01
		DANN	4.41E-03
BRCA2	15	PAFA	**4.59E-05**
		Eigen	1.41E-02
		CADD v1.3	1.01E-03
		GWAVA (Region Score)	9.65E-01
		GWAVA (TSS Score)	1.21E-01
		GWAVA (Unmatched Score)	5.44E-02
		FATHMM-MKL(Non-Coding Score)	6.97E-01
		FATHMM-MKL(Coding Score)	5.81E-01
		DANN	6.94E-02
CFTR	41	PAFA	4.16E-17
		Eigen	**3.31E-17**
		CADD v1.3	4.34E-13

		GWAVA (Region Score)	1.06E-04
		GWAVA (TSS Score)	1.48E-01
		GWAVA (Unmatched Score)	8.67E-02
		FATHMM-MKL(Non-Coding Score)	2.01E-14
		FATHMM-MKL(Coding Score)	8.60E-15
		DANN	1.58E-09
MLL2	92	PAFA	**1.07E-28**
		Eigen	**7.08E-47**
		CADD v1.3	4.82E-36
		GWAVA (Region Score)	5.97E-03
		GWAVA (TSS Score)	8.46E-02
		GWAVA (Unmatched Score)	6.54E-01
		FATHMM-MKL(Non-Coding Score)	1.82E-02
		FATHMM-MKL(Coding Score)	1.38E-18
		DANN	5.32E-06
TERT*	41	PAFA	**7.10E-07**
		Eigen	1.64E-01
		CADD v1.3	9.49E-03
		GWAVA (Region Score)	7.95E-02
		GWAVA (TSS Score)	7.91E-01
		GWAVA (Unmatched Score)	8.83E-01
		FATHMM-MKL(Non-Coding Score)	1.46E-01
		FATHMM-MKL(Coding Score)	2.89E-01
		DANN	6.56E-03

Table S4. Statistics of ten cancer-related variant sets from ICGC projects.

project key	country	project name	total number	noncoding common number	recurrent (common)	non-recurrent (common)	noncoding rare number	recurrent (rare)	non-recurrent (rare)
BLCA-CN	china	Bladder Urothelial carcinoma -CN	15,478	127	53	74	963	283	680
COCA-CN	china	Colorectal Cancer	399,862	74,685	12,955	61,730	242,564	47,680	194,884
ESCA-CN	china	Esophageal Cancer -CN	30,241	2,178	546	1,632	5,408	788	4620
PAEN-AU	Australia	Pancreatic Cancer Endocrine neoplasms-AU	144,516	3,794	447	3,347	129,975	7,298	122,677
BOCA-FR	France	Bone Cancer-Ewing Sarcoma-FR	36,607	1,436	166	1,270	31,632	1,242	30,390
EOPC-DE	Germany	Early Onset Prostate Cancer-DE	87,648	5,434	732	4,702	76,763	4,707	72,056
PAEN-IT	Italy	Pancreatic Endocrine neoplasms-IT	106,578	2,614	212	2,402	95,741	2,085	93,656
BTCA-JP	Japan	Biliary Tract Cancer-JP	66,751	1,999	451	1,548	25,388	3,250	22,138
THCA-SA	Saudi Arabia	Thyroid Cancer-SA	21,261	614	132	482	1,181	336	845
LAML-KR	South Korea	Acute Myeloid Leukemia-KR	137,216	7,761	2,665	5,096	111,088	42,900	68,188

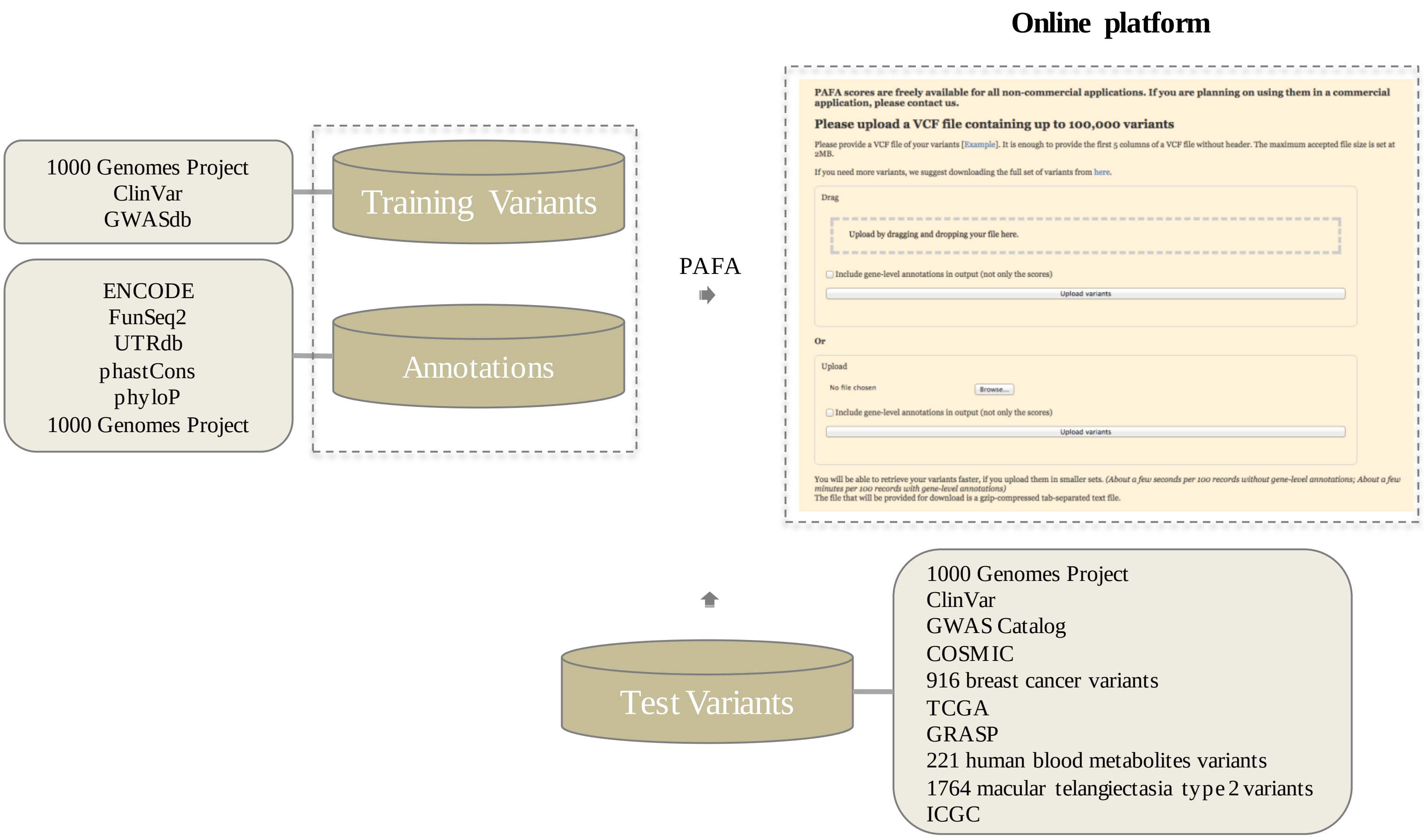

Figure S1. Genetic and genomic resources used in PAFA and their screenshots. PAFA contains variants used in the training stage and feature groups to annotate variants. Variants from multiple sources are used for testing the performance of PAFA.

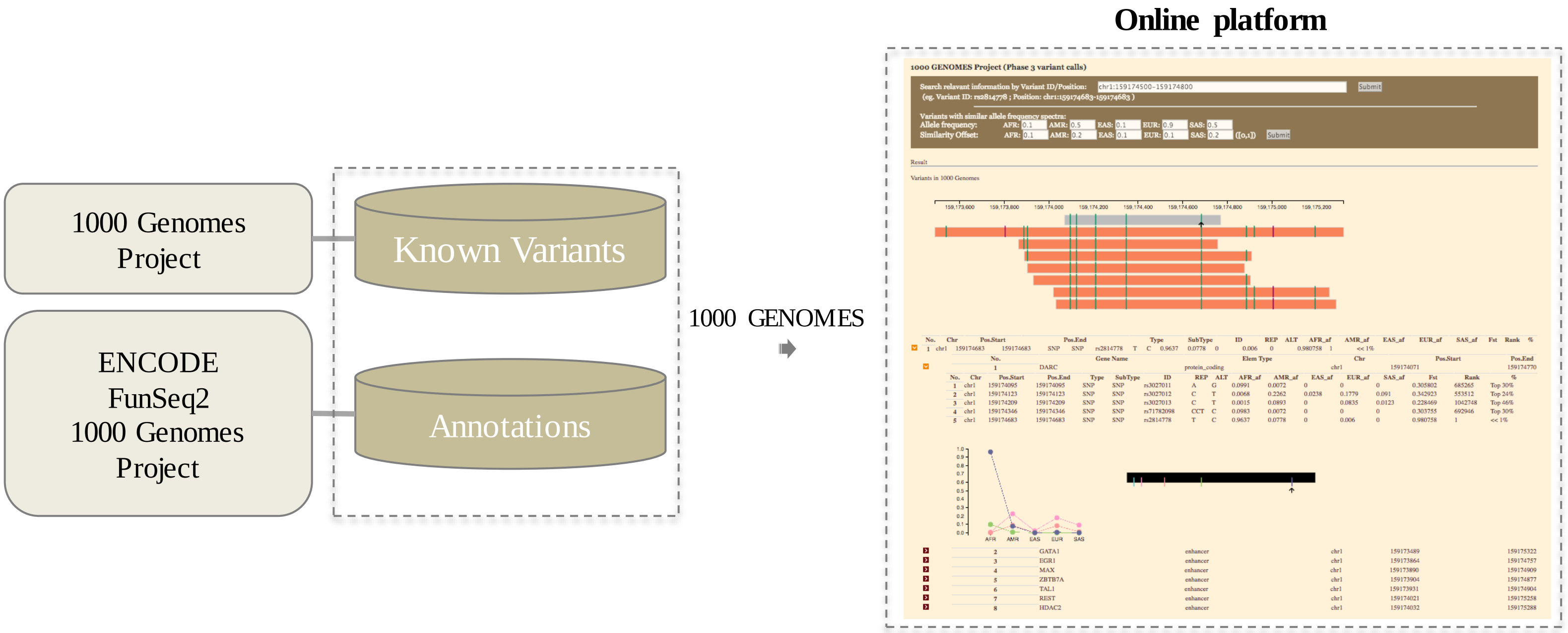

Figure S2. Genetic and genomic resources used in the 1000 GENOMES part of the PAFA online platform and their screenshots.

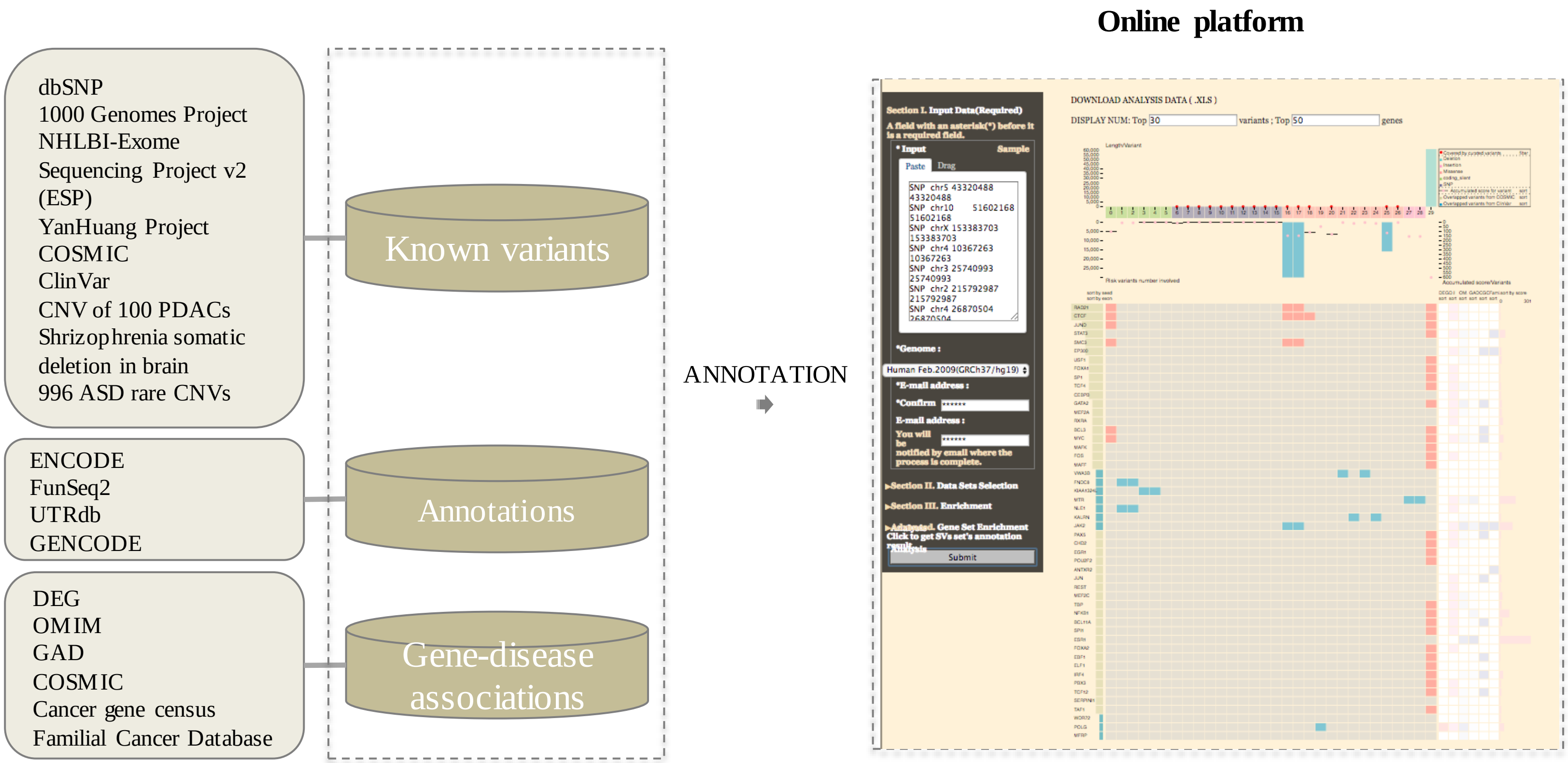

Figure S3. Genetic and genomic resources used in the ANNOTATION part of the PAFA online platform and their screenshots.

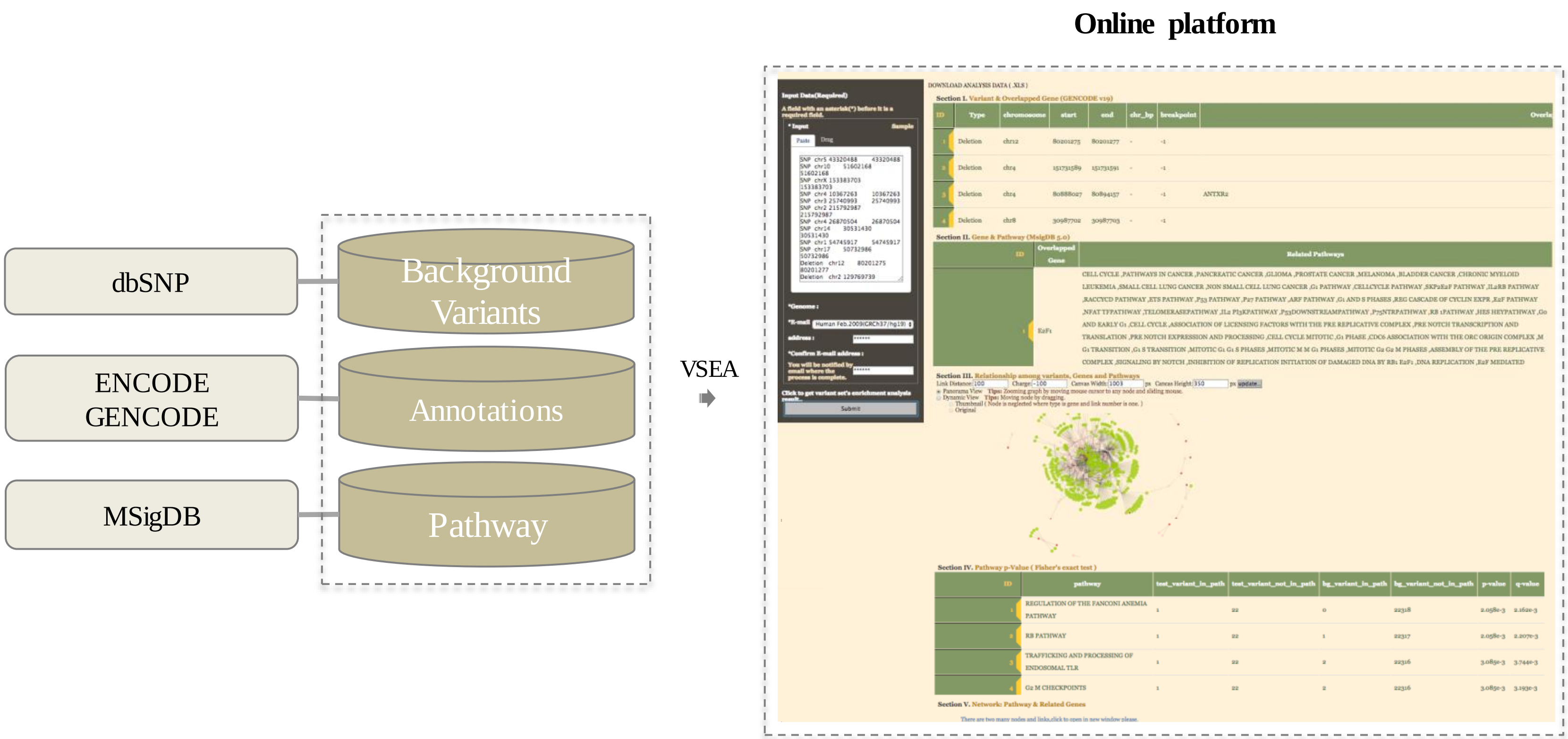

Figure S4. Genetic and genomic resources used in the VSEA part of the PAFA online platform and their screenshots.

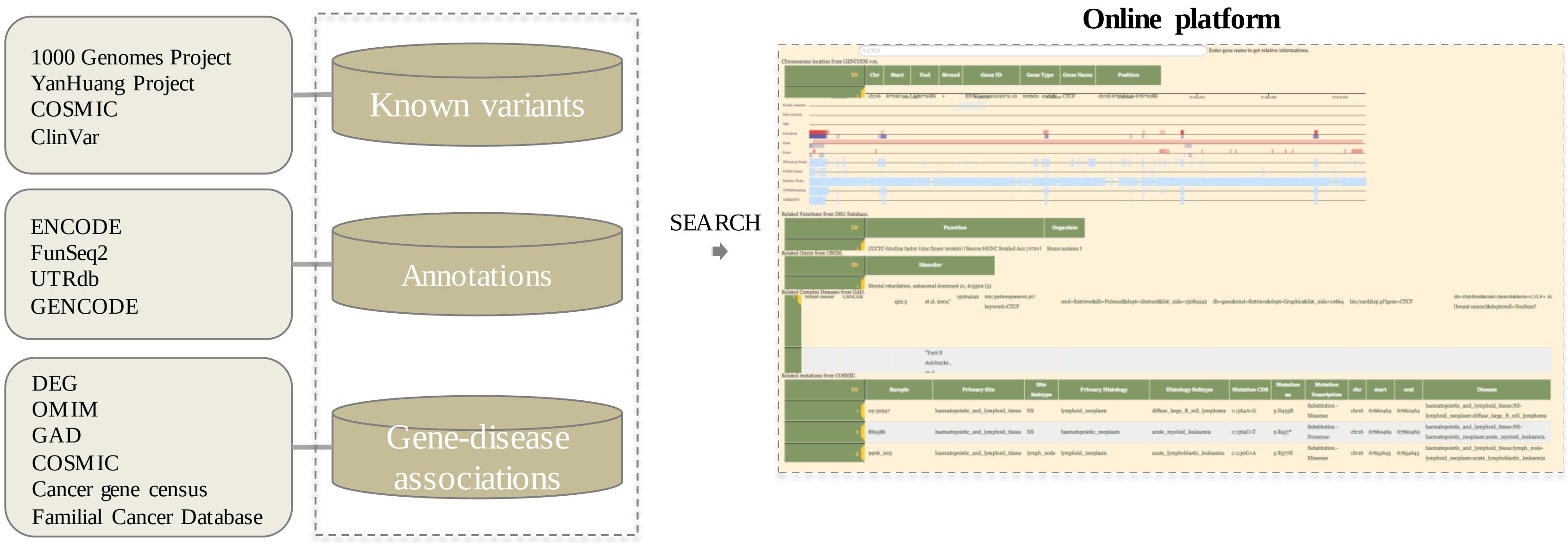

Figure S5. Genetic and genomic resources used in the SEARCH part of the PAFA online platform and their screenshots.

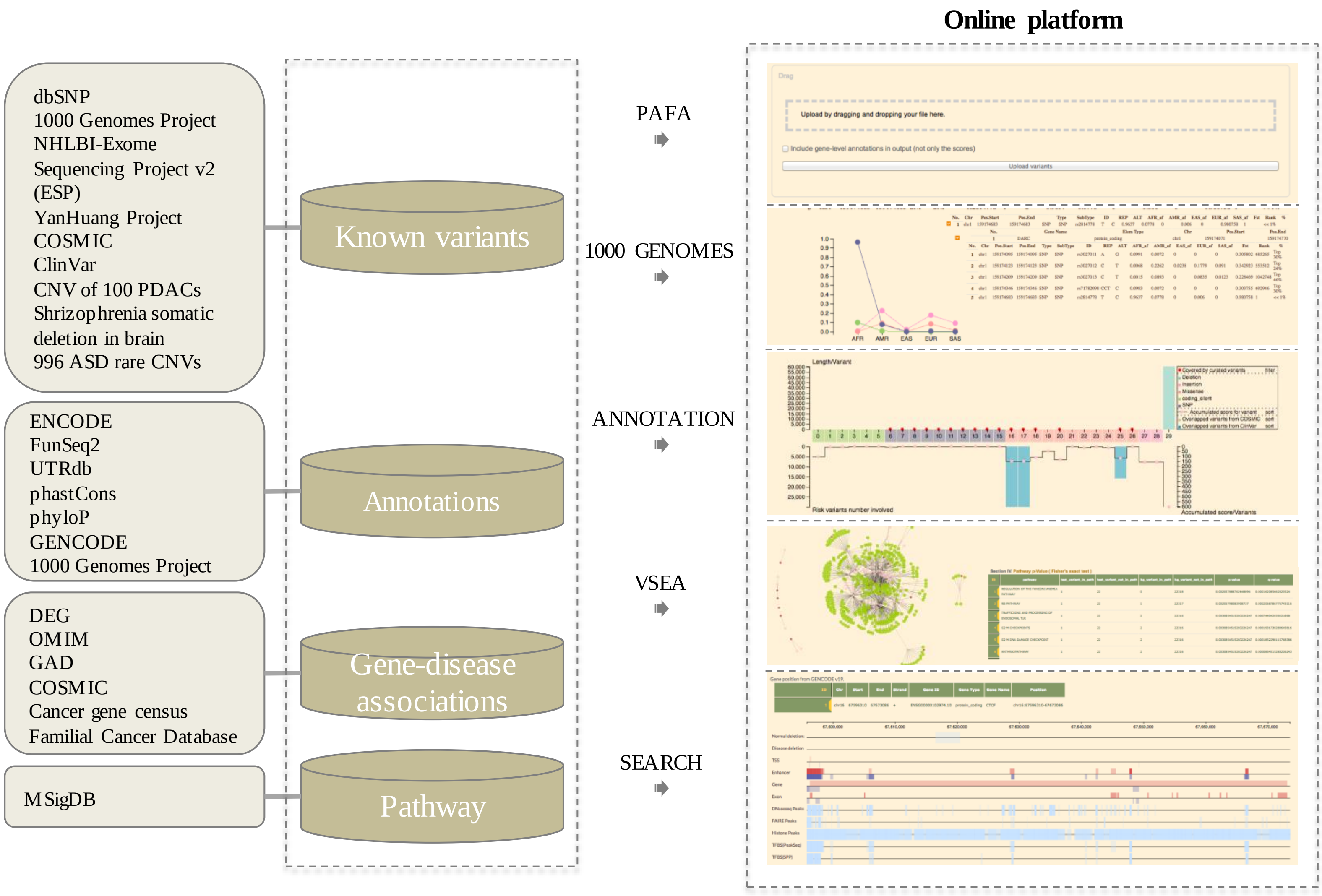

Figure S6. An integrated PAFA online platform for variant prioritization and functional annotation. Besides providing access to PAFA scores, the online platform includes the following functions: providing population-level information for variants found in 1000 Genomes, including FST index, allele frequency spectrum and associated genomic elements (**1000 GENOMES**), annotating variants with known risk variants, genomic annotations, diseases-associated genes and pathways recoded in curated databases (**ANNOTATION**), providing enrichment analysis on variant set (**VSEA**), displaying annotations from GENCODE, DEG database, OMIM, GAD and COSMIC by searching gene name (**SEARCH**).

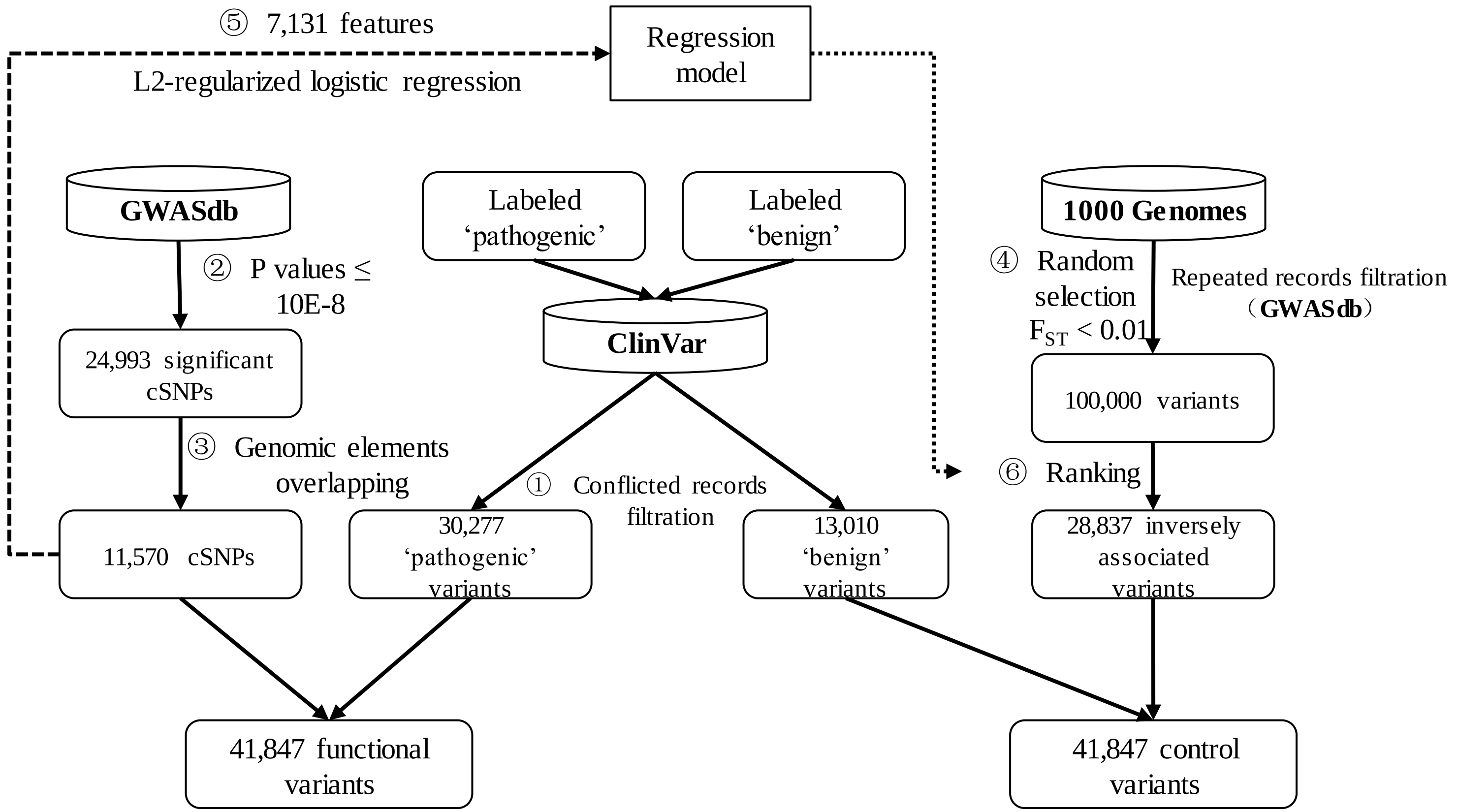

Figure S7. Flow chart of selecting and filtering training variants used in PAFA.

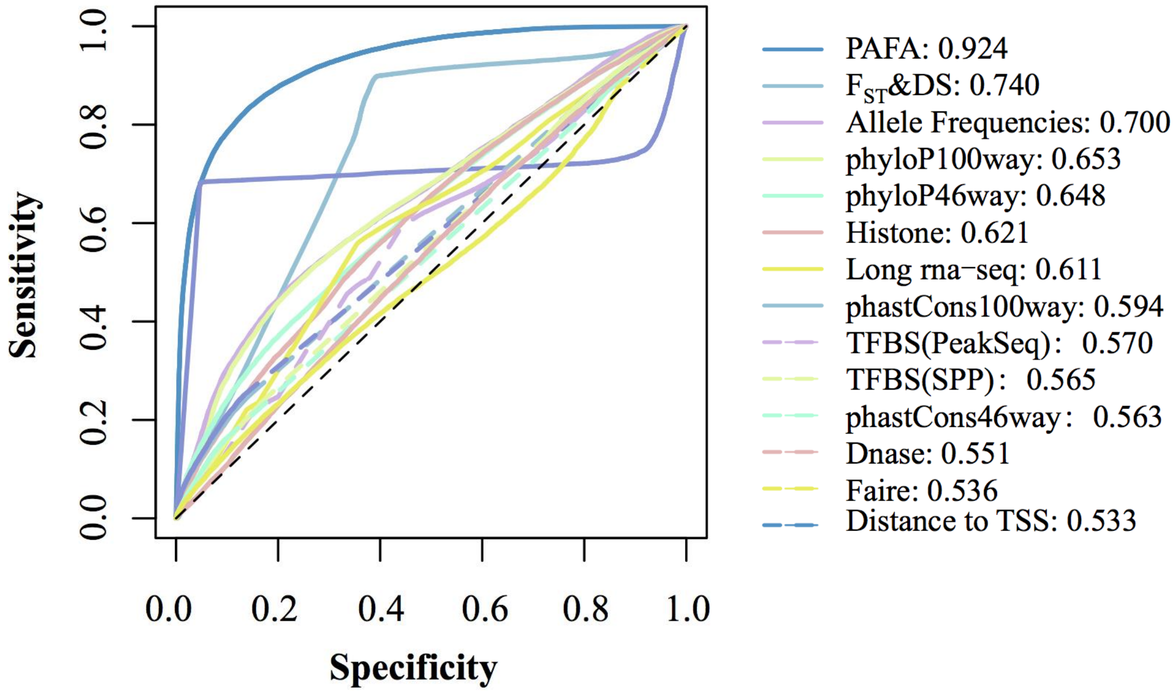

Figure S8. Ten-fold cross-validations are applied to evaluate the performance of features used in PAFA.

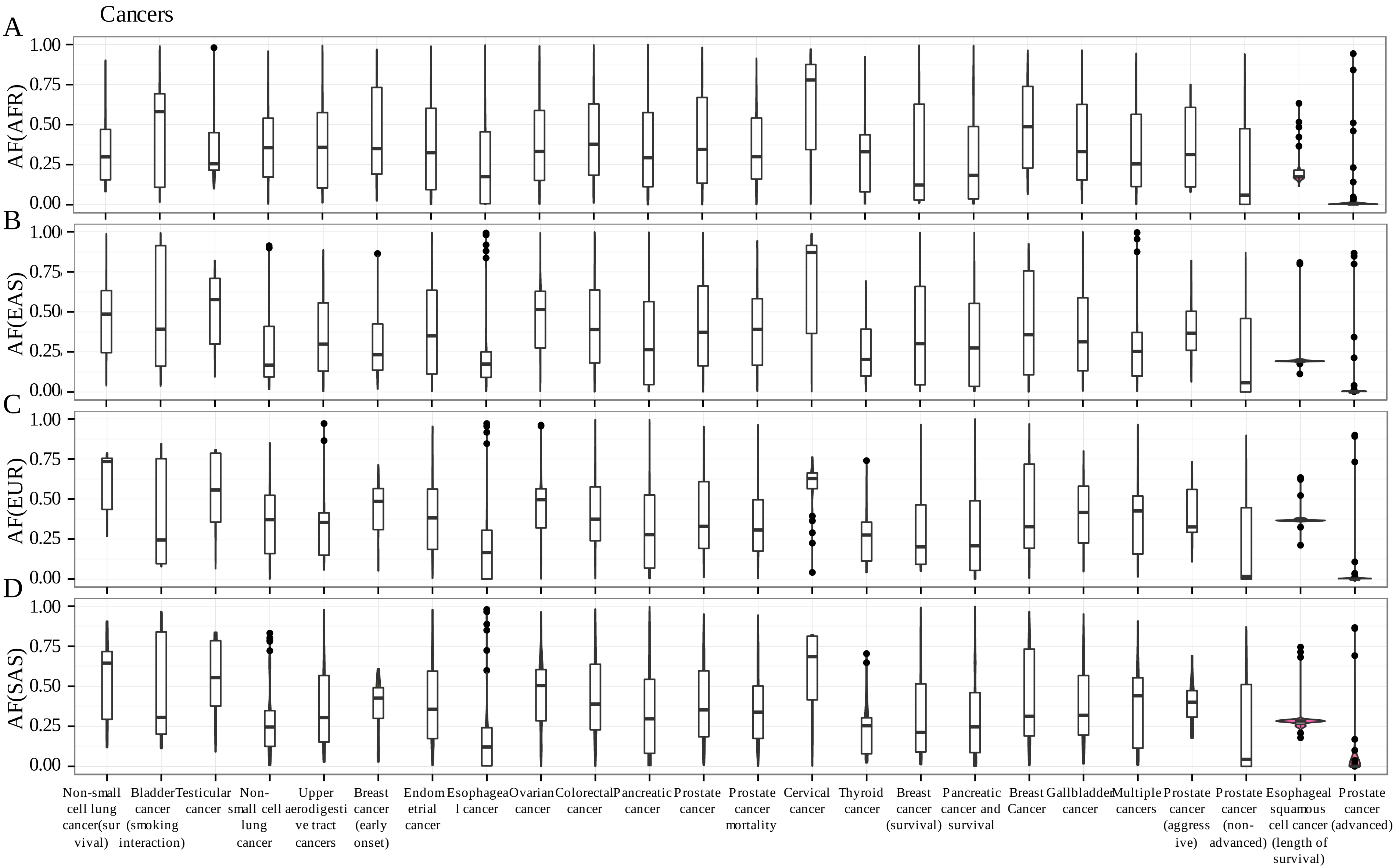

Figure S9. Distribution of allele frequencies for twenty-four cancer-associated variant sets from GWASdb among super populations. (**A**) Allele frequencies among African (AFR). (**B**) Allele frequencies among East Asian (EAS). (**C**) Allele frequencies among European (EUR). (**D**) Allele frequencies among South Asian (SAS).

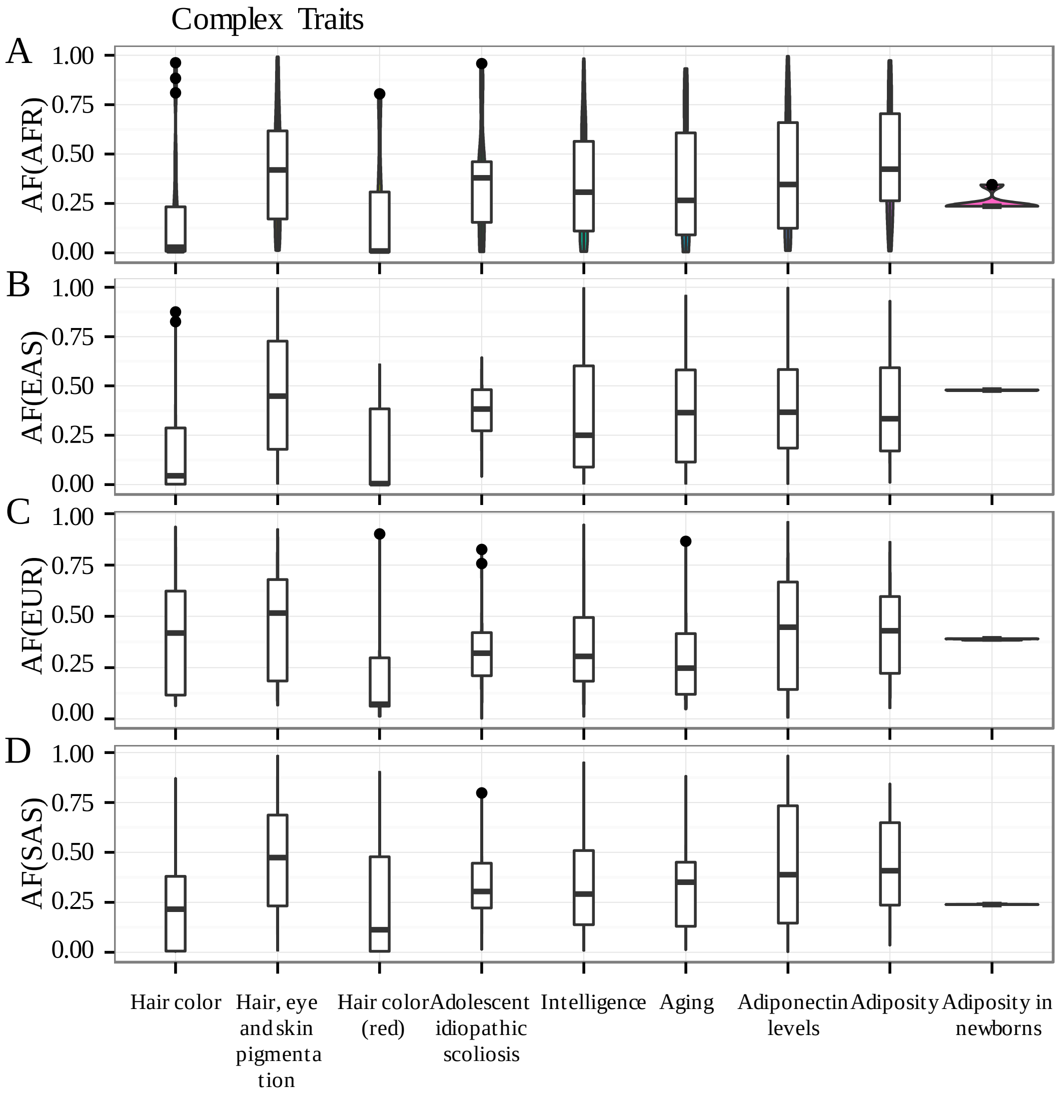

Figure S10. Distribution of allele frequencies for nine complex trait-associated variant sets from GWASdb among super populations. (**A**) Allele frequencies among African (AFR). (**B**) Allele frequencies among East Asian (EAS). (**C**) Allele frequencies among European (EUR). (**D**) Allele frequencies among South Asian
(SAS).

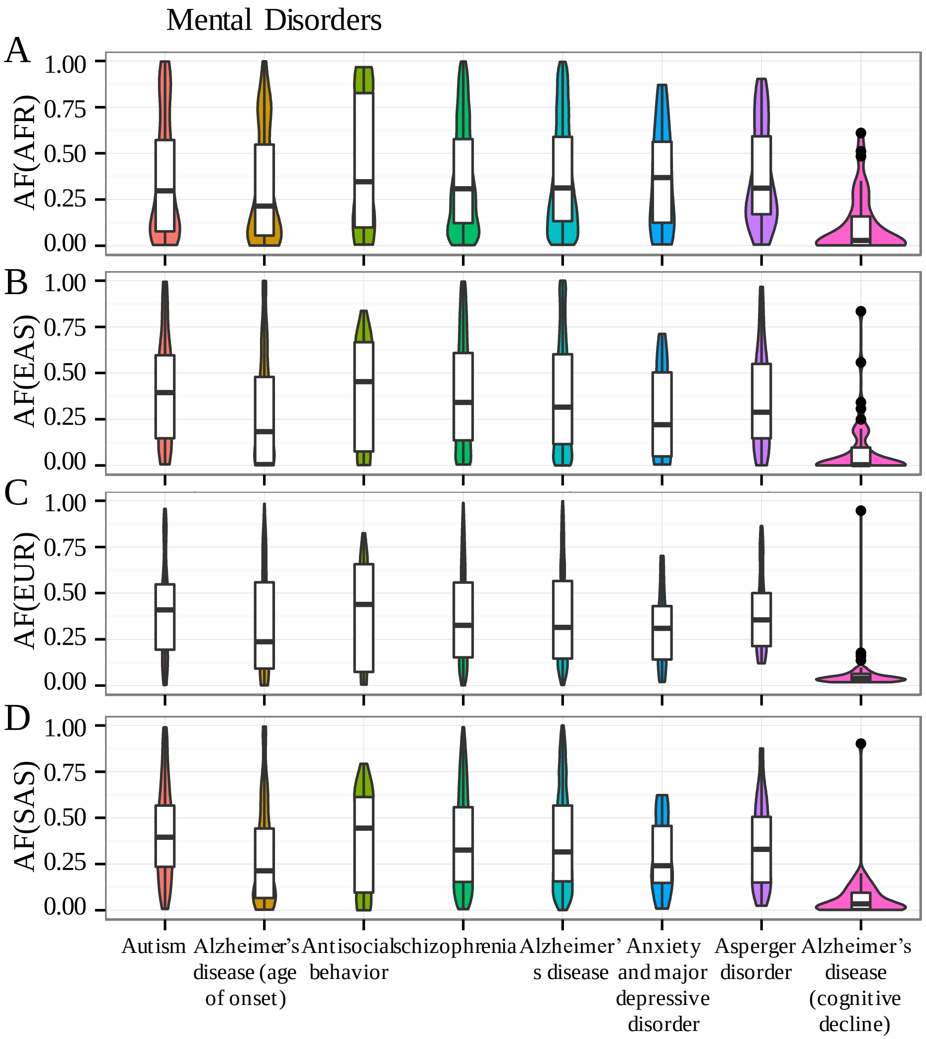

Figure S11. Distribution of allele frequencies for eight mental disorder-associated variant sets. (**A**) Allele frequencies among African (AFR). (**B**) Allele frequencies among East Asian (EAS). (**C**) Allele frequencies among European (EUR). (**D**) Allele frequencies among South Asian (SAS).

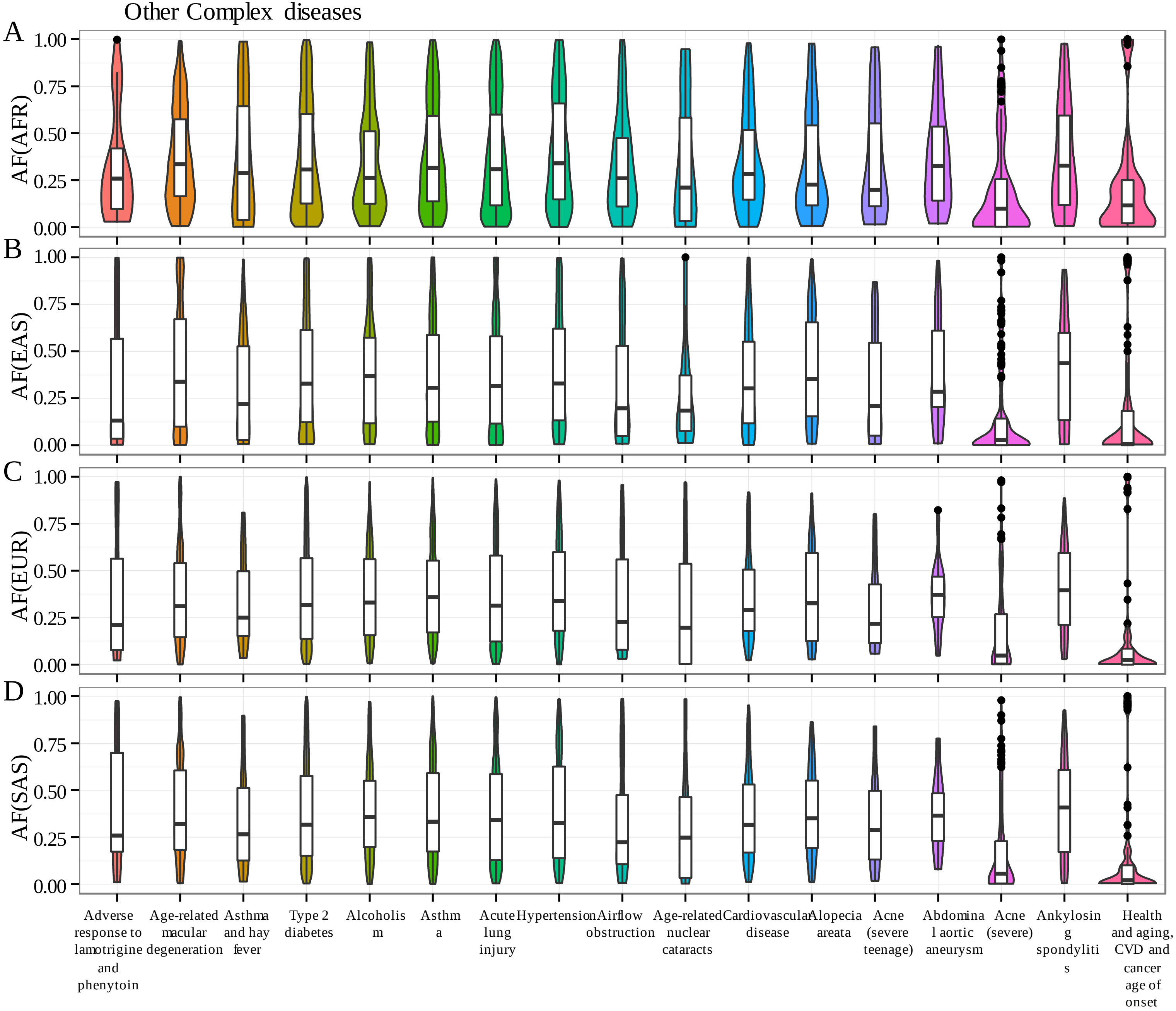

Figure S12. Distribution of allele frequencies for seventeen complex disease-associated variant sets. (**A**) Allele frequencies among African (AFR). (**B**) Allele frequencies among East Asian (EAS). (**C**) Allele frequencies among European (EUR). (**D**) Allele frequencies among South Asian (SAS).

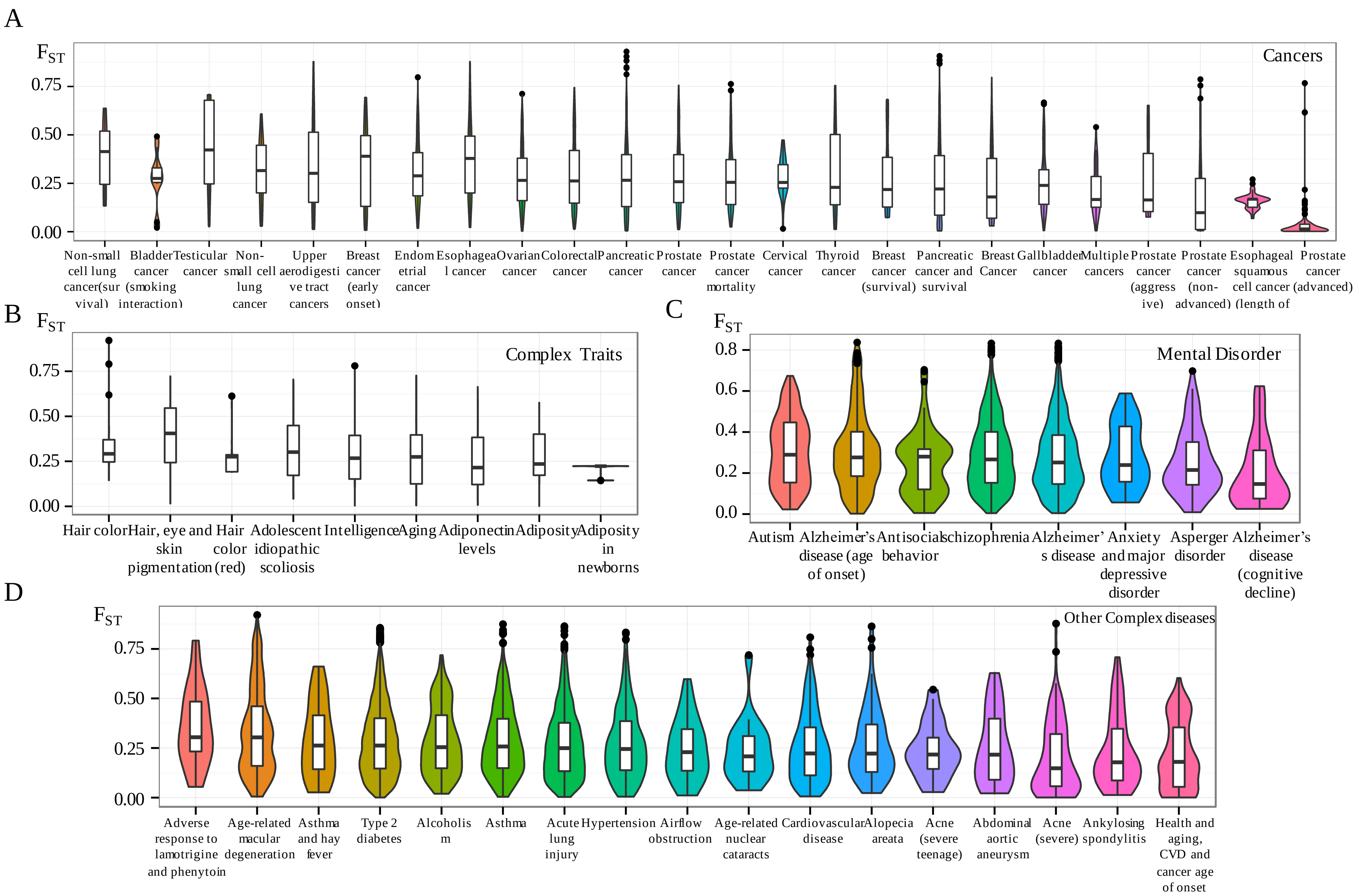

Figure S13. Distribution of FST values for variant sets associated with complex diseases and traits. (**A**) Cancer-associated variant sets. (**B**) Complex trait-associated variant sets. (**C**) Mental disorder-associated variant set. (**D**) Other complex disease-associated variant sets.

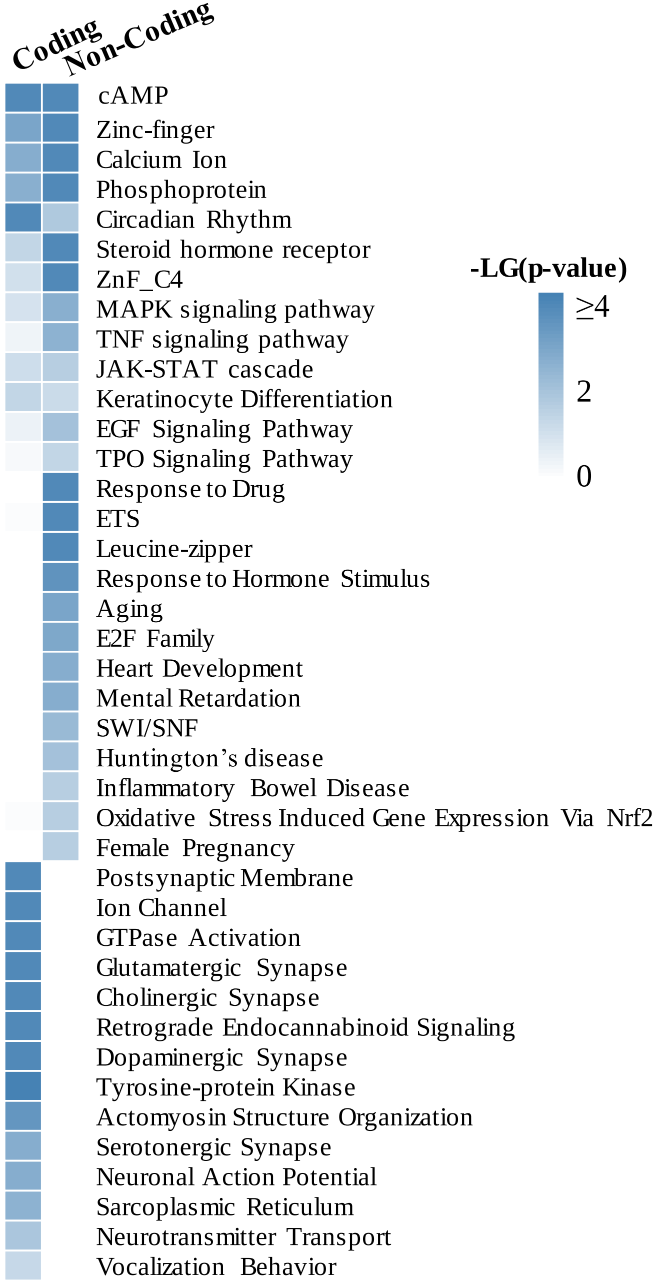

Figure S14. Enriched pathways of genes associated with coding and non-coding variants. DAVID was applied to analyze the enrichment of gene sets.

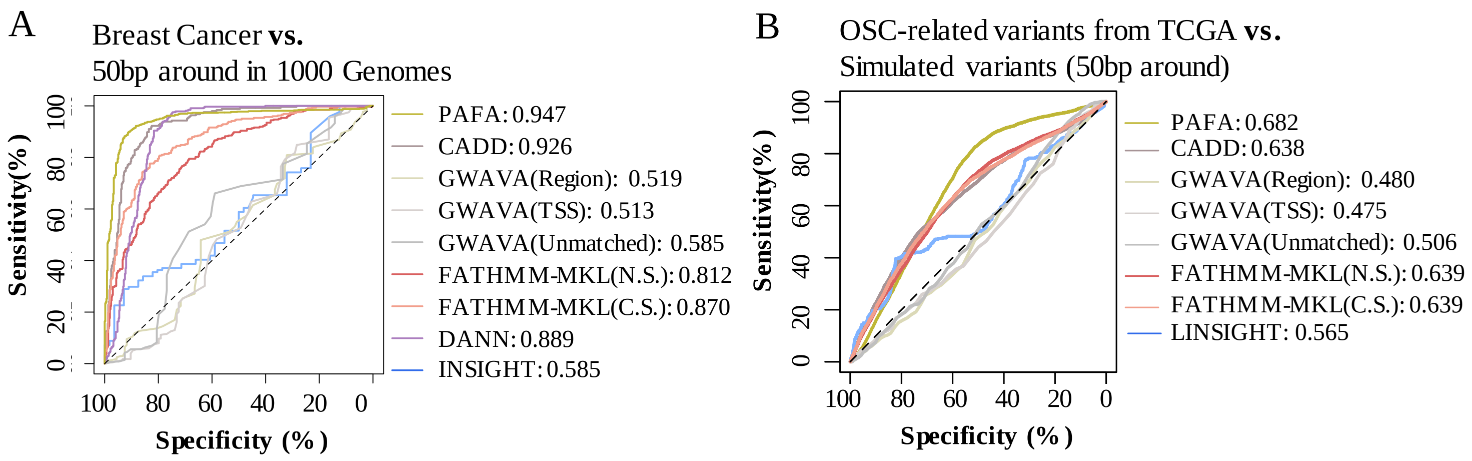

Figure S15. Sensitivity and specificity of tools in distinguishing coding risk variants from adjacent variants. Receiver operating characteristics (ROC) curves are exhibited. The dashed line indicates random chance. The value of the area under the curve (AUC) is calculated for each score. (**A**) Prioritization of 916 possible driver variants associated with breast cancer from 474 adjacent variants (50 bp upstream and downstream) from 1000 Genomes. (**B**) Prioritization of 6133 variants associated with ovarian serous cystadenocarcinoma (OSC) from 6133 simulated noncoding rare variants (50 bp upstream and downstream). Such analyses were repeated 10 times and the average AUC values were shown.

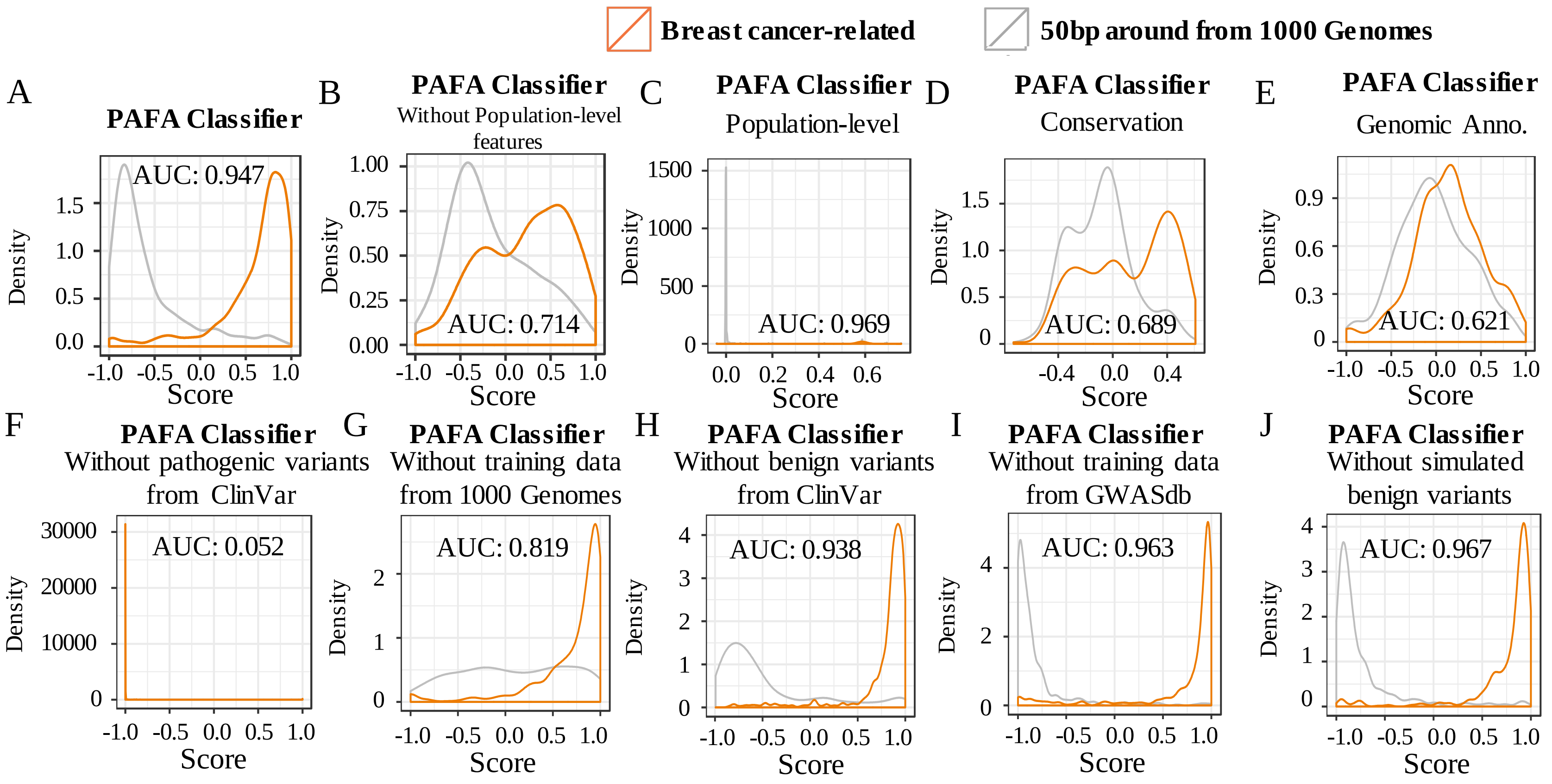

Figure S16. Distributions of PAFA scores for breast cancer-related variants and adjacent variants from the 1000 Genomes. (**A**) The PAFA classifier. (**B**) The PAFA classifier constructed based on evolutionary conservation and genomic annotation features. (**C**) The PAFA classifier constructed based on population differentiation features. (**D**) The PAFA classifier constructed based on evolutionary conservation features. (**E**) The PAFA classifier constructed based on genomic annotation features. (**F**) The PAFA classifier constructed without pathogenic variants from ClinVar. (**G**) The PAFA classifier constructed without training data from 1000 Genomes. (**H**) The PAFA classifier constructed without benign variants from ClinVar. (**I**) The PAFA classifier constructed without training data from GWASdb. (**J**) The PAFA classifier constructed without simulated benign variants.

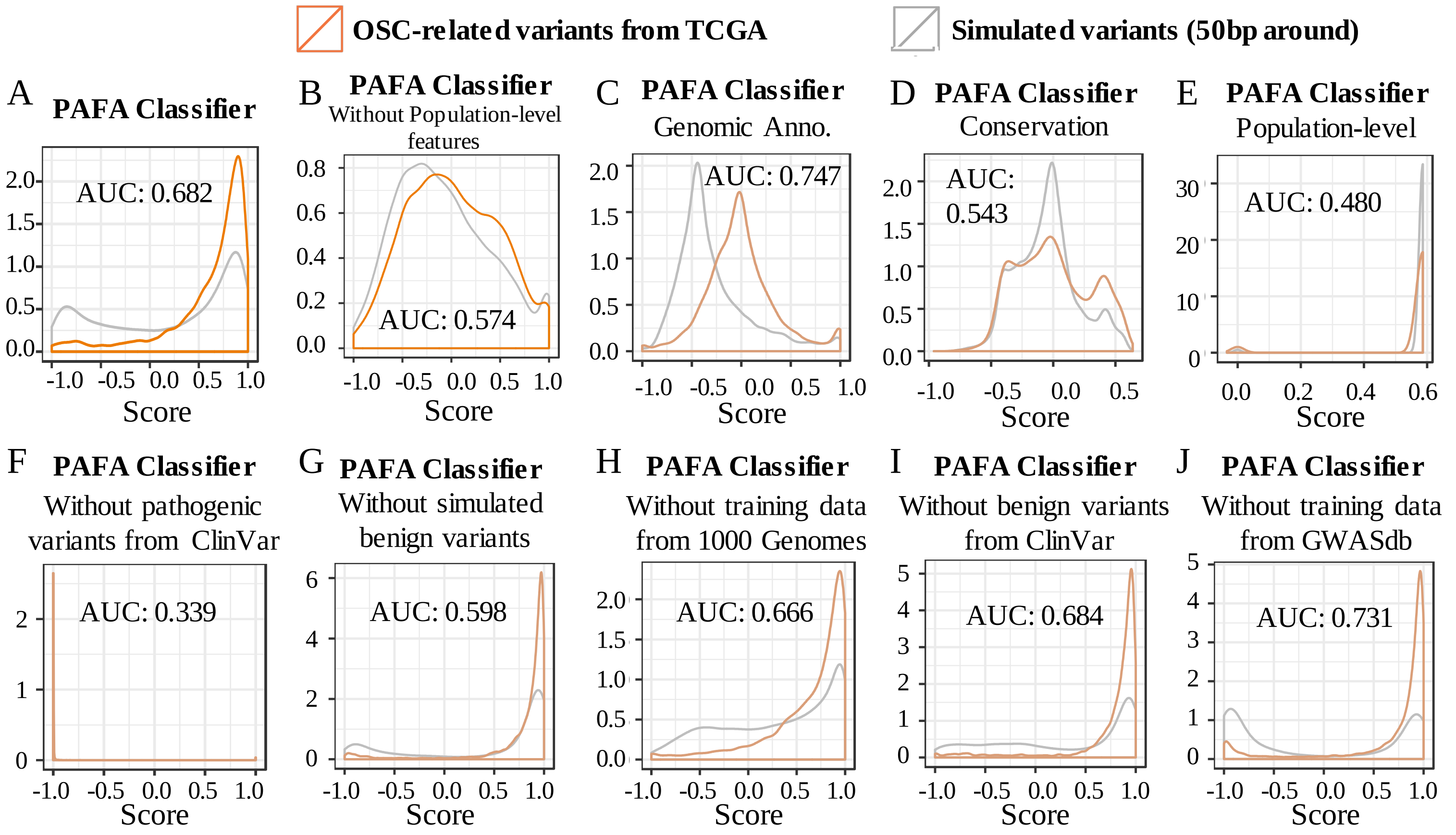

Figure S17. Distributions of PAFA scores for OSC-related variants from TCGA and simulated noncoding rare variants. (**A**) The PAFA classifier. (**B**) The PAFA classifier constructed based on evolutionary conservation and genomic annotation features. (**C**) The PAFA classifier constructed based on genomic annotation features. (**D**) The PAFA classifier constructed based on evolutionary conservation features. (**E**) The PAFA classifier constructed based on population differentiation features. (**F**) The PAFA classifier constructed without pathogenic variants from ClinVar. (**G**) The PAFA classifier constructed without simulated benign variants. (**H**) The PAFA classifier constructed without training data from 1000 Genomes. (**I**) The PAFA classifier constructed without benign variants from ClinVar. (**J**) The PAFA classifier constructed without training data from GWASdb.

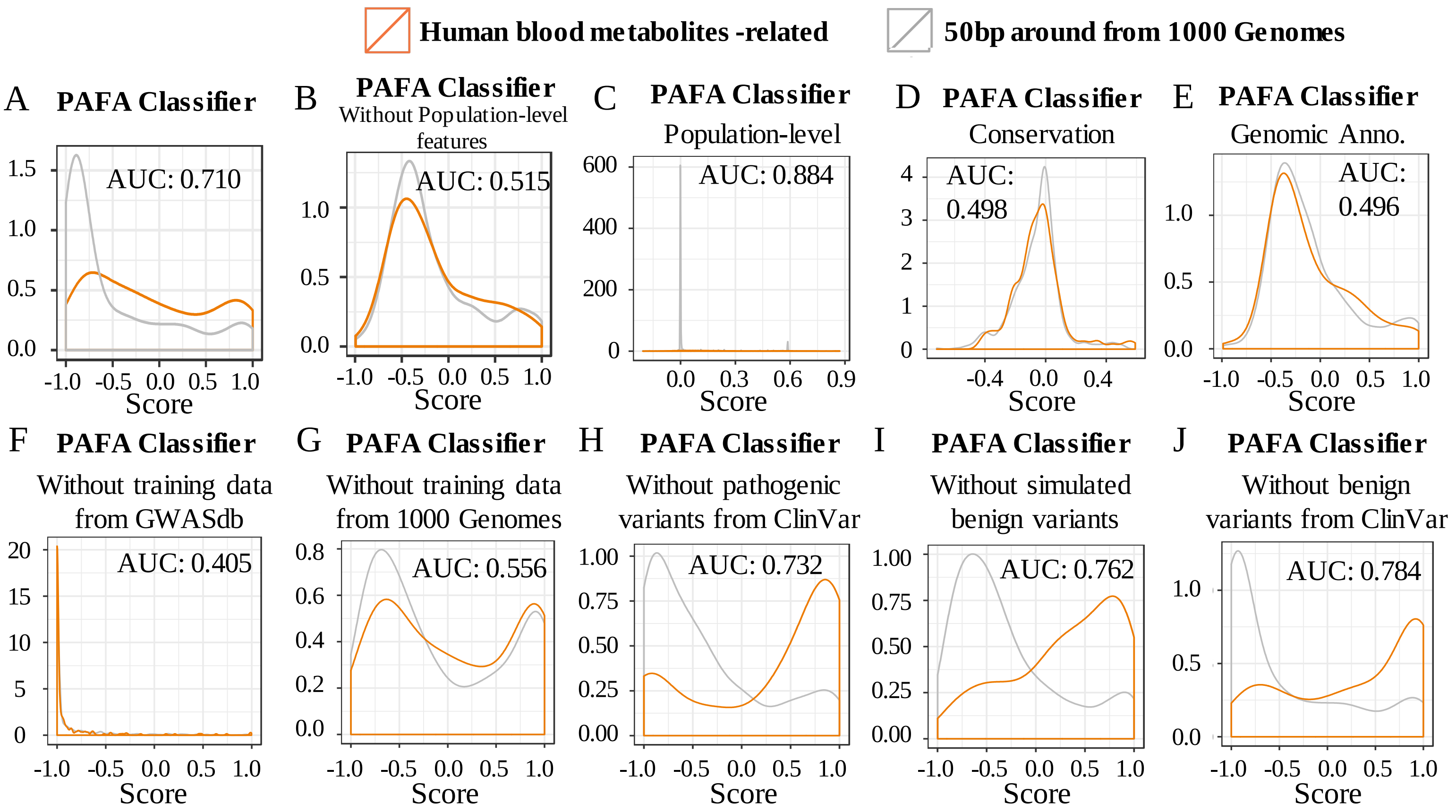

Figure S18. Distributions of PAFA scores for human blood metabolites-related variants and adjacent variants from 1000 Genomes. (**A**) The PAFA classifier. (**B**) The PAFA classifier constructed based on evolutionary conservation and genomic annotation features. (**C**) The PAFA classifier constructed based on population differentiation features. (**D**) The PAFA classifier constructed based on evolutionary conservation features. (**E**) The PAFA classifier constructed based on genomic annotation features. (**F**) The PAFA classifier constructed without training data from GWASdb. (**G**) The PAFA classifier constructed without training data from 1000 Genomes. (**H**) The PAFA classifier constructed without pathogenic variants from ClinVar. (**I**) The PAFA classifier constructed without simulated benign variants. (**J**) The PAFA classifier constructed without benign variants from ClinVar.

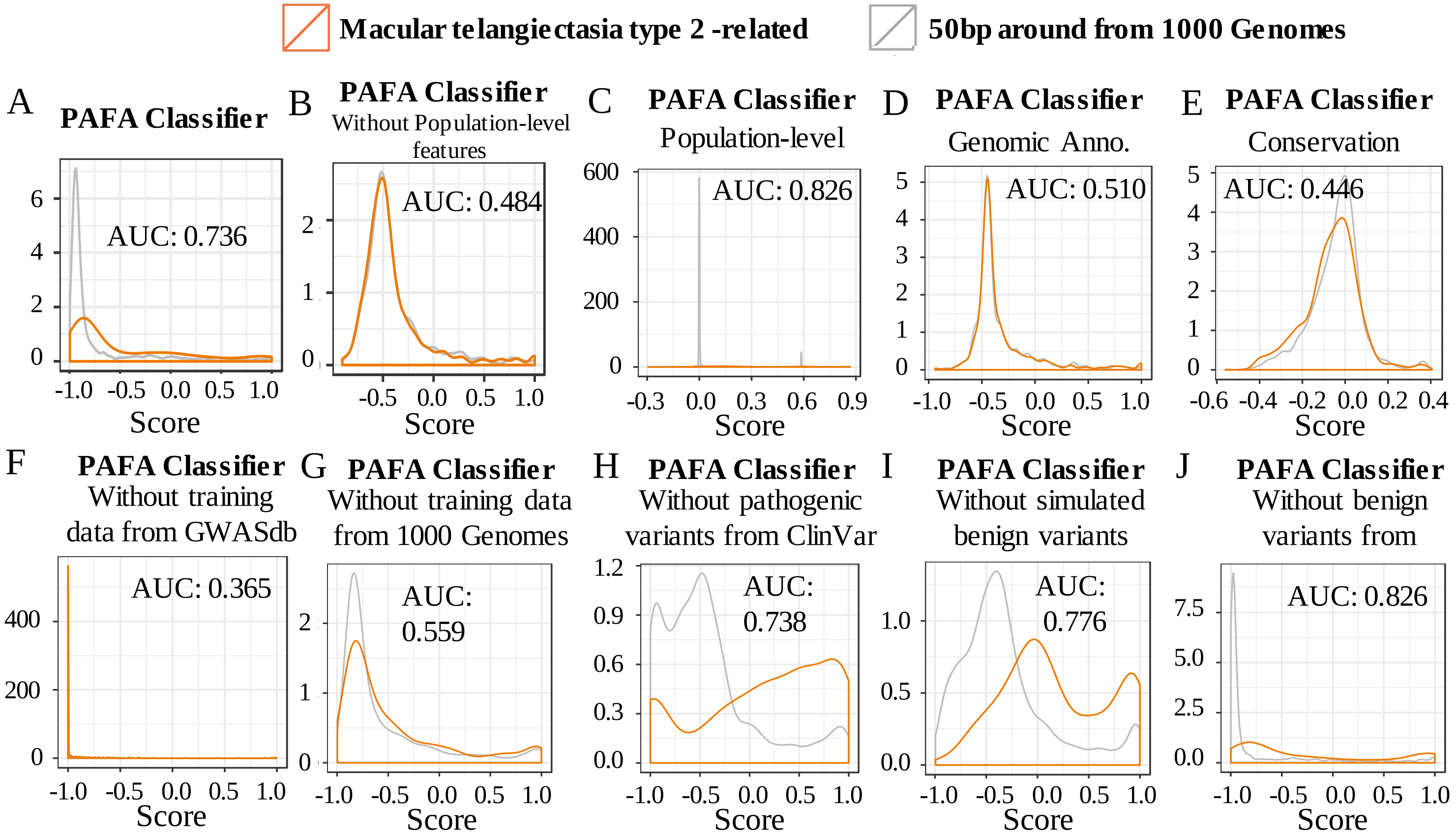

Figure S19. Distributions of PAFA scores for macular telangiectasia type 2-related variants and adjacent variants from 1000 Genomes. (**A**) The PAFA classifier. (**B**) The PAFA classifier constructed based on evolutionary conservation and genomic annotation features. (**C**) The PAFA classifier constructed based on population differentiation features. (**D**) The PAFA classifier constructed based on genomic annotation features. (**E**) The PAFA classifier constructed based on evolutionary conservation features. (**F**) The PAFA classifier constructed without training data from GWASdb. (**G**) The PAFA classifier constructed without training data from 1000 Genomes. (**H**) The PAFA classifier constructed without pathogenic variants from ClinVar. (**I**) The PAFA classifier constructed without simulated benign variants. (**J**) The PAFA classifier constructed without benign variants from ClinVar.

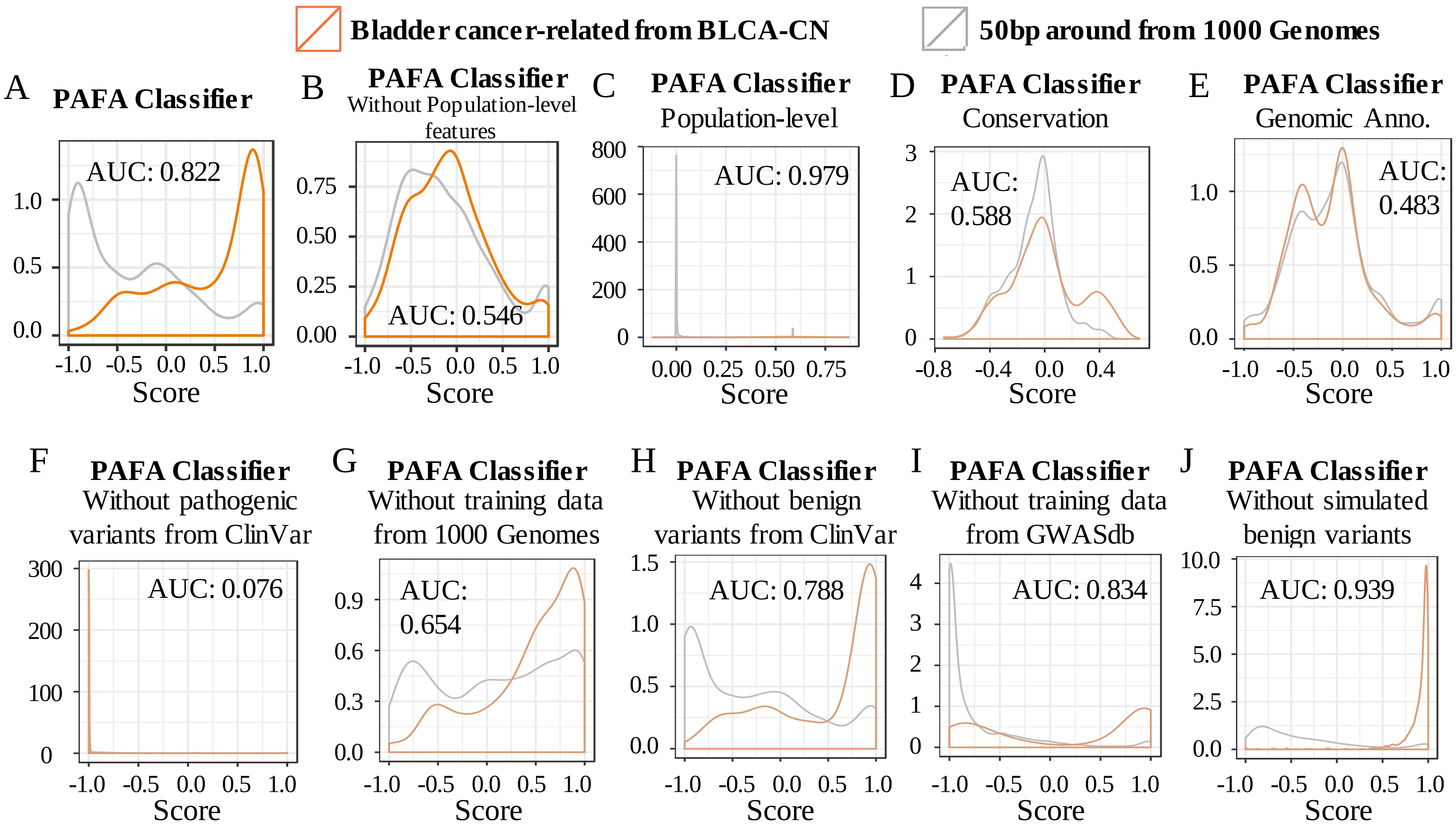

Figure S20. Distributions of PAFA scores for bladder cancer-related variants and adjacent variants from 1000 Genomes. (**A**) The PAFA classifier. (**B**) The PAFA classifier constructed based on evolutionary conservation and genomic annotation features. (**C**) The PAFA classifier constructed based on population differentiation features. (**D**) The PAFA classifier constructed based on evolutionary conservation features. (**E**) The PAFA classifier constructed based on genomic annotation features. (**F**) The PAFA classifier constructed without pathogenic variants from ClinVar. (**G**) The PAFA classifier constructed without training data from 1000 Genomes. (**H**) The PAFA classifier constructed without benign variants from ClinVar. (**I**) The PAFA classifier constructed without training data from GWASdb. (**J**) The PAFA classifier constructed without simulated benign variants
